# Both direct and indirect suppression of MCL1 synergizes with BCLXL inhibition in preclinical models of gastric cancer

**DOI:** 10.1038/s41419-025-07481-8

**Published:** 2025-03-12

**Authors:** Li-Ping Zhang, Yu-Min Wei, Ming-Jie Luo, Shu-Yue Ren, Xiang-Wen Zhan, Chao Wang, Ze-Feng Li, Rui-Min Zhu, Shuo Yan, Yu Cheng, Jia-Li Xu, Xing-Jiu Yang, Ke-Lei Du, Jin-Qing Wang, Guan-nan Zhang, De-Xiao Du, Ran Gao, Dong-Bing Zhao, Jia-Nan Gong

**Affiliations:** 1 National Center of Technology Innovation for Animal Model, National Human Diseases Animal Model Resource Center, NHC Key Laboratory of Human Disease Comparative Medicine, The Institute of Laboratory Animal Sciences, Chinese Academy of Medical Sciences & Peking Union Medical College, Beijing, China; 2 State Key Laboratory of Ophthalmology, Zhongshan Ophthalmic Center, Sun Yat-Sen University, Guangzhou, China; 3 Department of Pancreatic and Gastric Surgical Oncology, National Cancer Center/National Clinical Research Center for Cancer/Cancer Hospital, Chinese Academy of Medical Sciences and Peking Union Medical College, Beijing, China; 4 Department of Gastrointestinal Surgery, The Second Hospital of Shandong University, Jinan, China; 5 Division of Colorectal Surgery, Department of General Surgery, Peking Union Medical College Hospital, Chinese Academy of Medical Sciences & Peking Union Medical College, Beijing, China; 6 Department of general surgery, Capital Medical University Affiliated Beijing Shijitan Hospital, Beijing, China

**Keywords:** Targeted therapies, Apoptosis

## Abstract

Despite the progress of treatment in gastric cancer (GC), the overall outcomes remain poor in patients with advanced diseases, underscoring the urgency to develop more effective treatment strategies. BH3-mimetic drugs, which inhibit the pro-survival BCL2 family proteins, have demonstrated great therapeutic potential in cancer therapy. Although previous studies have implicated a role of targeting the cell survival pathway in GC, the contribution of different pro-survival BCL2 family proteins in promoting survival and mediating resistance to current standard therapies in GC remains unclear. A systematic study to elucidate the hierarchy of these proteins using clinically more relevant GC models is essential to identify the most effective therapeutic target(s) and rational combination strategies for improving GC therapy. Here, we provide evidence from both in vitro and in vivo studies using a broad panel of GC cell lines, tumoroids, and xenograft models to demonstrate that BCLXL and MCL1, but not other pro-survival BCL2 family proteins, are crucial for GC cells survival. While small molecular inhibitors of BCLXL or MCL1 exhibited some single-agent activity, their combination sufficed to cause maximum killing. However, due to the unsolved cardiotoxicity associated with direct MCL1 inhibitors, finding combinations of agents that indirectly target MCL1 and enable the reduction of doses of BCLXL inhibitors while maintaining their anti-neoplastic effects is potentially a feasible approach for the further development of these compounds. Importantly, inhibiting BCLXL synergized significantly with anti-mitotic and HER2-targeting drugs, leading to enhanced anti-tumour activity with tolerable toxicity in preclinical GC models. Mechanistically, anti-mitotic chemotherapies induced MCL1 degradation via the ubiquitin-proteasome pathway mainly through FBXW7, whereas HER2-targeting drugs suppressed MCL1 transcription via the STAT3/SRF axis. Moreover, co-targeting STAT3 and BCLXL also exhibited synergistic killing, extending beyond HER2-amplified GC. Collectively, our results provide mechanistic rationale and pre-clinical evidence for co-targeting BCLXL and MCL1 (both directly and indirectly) in GC.

**Figure:**
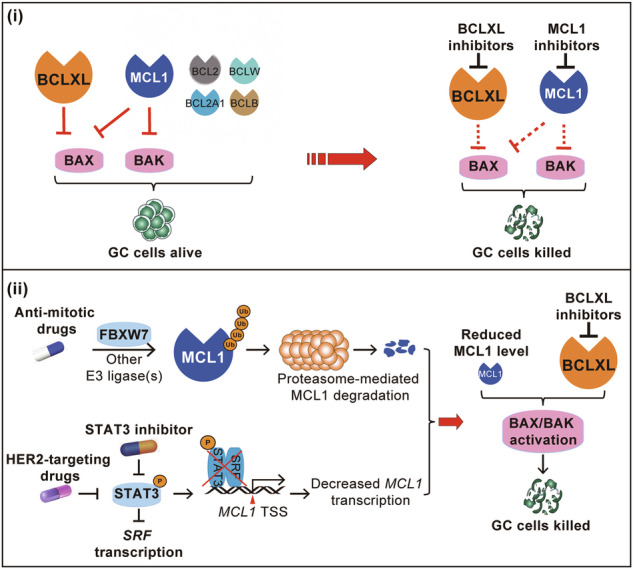
(**i**) Gastric cancer cells rely on BCLXL and, to a lesser degree, on MCL1 for survival. The dual inhibition of BCLXL and MCL1 with small molecular inhibitors acts synergistically to kill GC cells, regardless of their TCGA molecular subtypes or the presence of poor prognostic markers. While the effect of S63845 is mediated by both BAX and BAK in most cases, BAX, rather than BAK, acts as the primary mediator of BCLXLi in GC cells. (**ii**) Inhibiting BCLXL significantly synergizes with anti-mitotic and HER2-targeting drugs, leading to enhanced anti-tumour activity with tolerable toxicity in preclinical GC models. Mechanistically, anti-mitotic chemotherapies induce MCL1 degradation via the ubiquitin-proteasome pathway mainly through FBXW7, whereas HER2-targeting drugs suppress *MCL1* transcription via the STAT3/SRF axis. The combination of the STAT3 inhibitor and BCLXL inhibitor also exhibits synergistic killing, extending beyond HER2-amplified GC.

(**i**) Gastric cancer cells rely on BCLXL and, to a lesser degree, on MCL1 for survival. The dual inhibition of BCLXL and MCL1 with small molecular inhibitors acts synergistically to kill GC cells, regardless of their TCGA molecular subtypes or the presence of poor prognostic markers. While the effect of S63845 is mediated by both BAX and BAK in most cases, BAX, rather than BAK, acts as the primary mediator of BCLXLi in GC cells. (**ii**) Inhibiting BCLXL significantly synergizes with anti-mitotic and HER2-targeting drugs, leading to enhanced anti-tumour activity with tolerable toxicity in preclinical GC models. Mechanistically, anti-mitotic chemotherapies induce MCL1 degradation via the ubiquitin-proteasome pathway mainly through FBXW7, whereas HER2-targeting drugs suppress *MCL1* transcription via the STAT3/SRF axis. The combination of the STAT3 inhibitor and BCLXL inhibitor also exhibits synergistic killing, extending beyond HER2-amplified GC.

## Introduction

Gastric cancer ranks as the fifth most common and deadliest cancer globally, according to GLOBOCAN 2022 [1]. Approximately 60% of patients present with locally advanced or metastatic disease at diagnosis, and the outcomes for these patients remain very poor, with a median survival time of only 12–15 months [2, 3]. Nearly all patients with advanced diseases will progress after first-line therapy or present with primary refractory disease. Therefore, there is an unmet need to identify more effective treatment strategies for advanced GC patients.

In this context, targeting the BCL2-regulated intrinsic apoptosis pathway may be a promising complementary approach. BH3-mimetic drugs are a class of small molecular inhibitors acting to block one or more of the pro-survival BCL2 family proteins, thus allowing the activation of BAX and BAK to drive apoptosis [4]. Clinically, the most advanced of these BH3 mimetics is venetoclax, a selective inhibitor of BCL2, which has received approval by many regulatory authorities worldwide for treating chronic lymphocytic leukemia (CLL) and acute myeloid leukemia (AML) [5, 6]. To date, the clinical application of BH3-mimetic drugs in GC is barely explored. AT101 is a levorotatory enantiomer of gossypol acetic acid, which was reported to show strong anti-tumor activity in gastroesophageal cancers (GECs) [7]. However, the anti-tumor activity of AT101 in GECs relies primarily on targeting the cancer stem cells pathway, which remains active when BCL2 is downregulated [7]. Substantial data have suggested that this compound is highly unspecific and does not directly inhibit BCL2 or BCLXL, with its cytotoxic activity being independent of BAX and BAK [8–11]. Clinical development of AT-101 was underway but has now been halted.

Previous studies have implicated potential roles of pro-survival BCL2 family proteins in GC. The *BCL2L1* gene, which encodes BCLXL, is amplified in 2.7–10.7% of GC cases [12, 13]. Our study, along with others’, has reported that targeting BCLXL is effective to induce apoptosis in some GC cell lines [12–18]. However, the association between *BCL2L1* amplification and susceptibility to BCLXL inhibition remains elusive, with controversial results reported [12, 13]. Early studies using siRNAs or antisense oligonucleotides to target *MCL1* suggested that MCL1 may also play a role in GC cells survival [19–22]. Roles for BCL2 and BCLW were also proposed [18, 23–26]. Dysregulation of apoptosis was also linked to drug resistance in GC [27]. However, these prior studies only focused on individual genes and were conducted using a limited number of GC cell lines. A systematic study using clinically more relevant GC models to elucidate the hierarchy of these pro-survival BCL2 family proteins in promoting survival and mediating drug resistance in GC is lacking.

Here, we provide evidence from both in vitro and in vivo experiments, employing a wide range of GC cell lines, patient-derived tumor organoids (PDOs) as well as CDX and PDX xenograft models, to demonstrate that BCLXL and MCL1 are key for GC cell survival. While targeting BCLXL or MCL1 exhibited some single-agent activity, their combined inhibition sufficed to cause maximum killing. Given the unsolved cardiotoxicity associated with direct MCL1 inhibitors, we next searched for agents that indirectly target MCL1 and identified three classes of drugs, including anti-mitotic chemotherapies, HER2-targeting drugs, and the STAT3 inhibitor. We demonstrated that these drugs downregulate MCL1 activity via distinct mechanisms and synergize significantly with the BCLXL inhibitor, leading to enhanced anti-tumor activity with tolerable toxicity in preclinical GC models. Our study therefore provides mechanistic rationale and pre-clinical evidence for co-targeting BCLXL and MCL1 (both directly and indirectly) in GC.

## Results

### Response of a panel of GC cell lines representing the four TCGA molecular subtypes to well-validated BH3-mimetic compounds

**Fig. 1:**
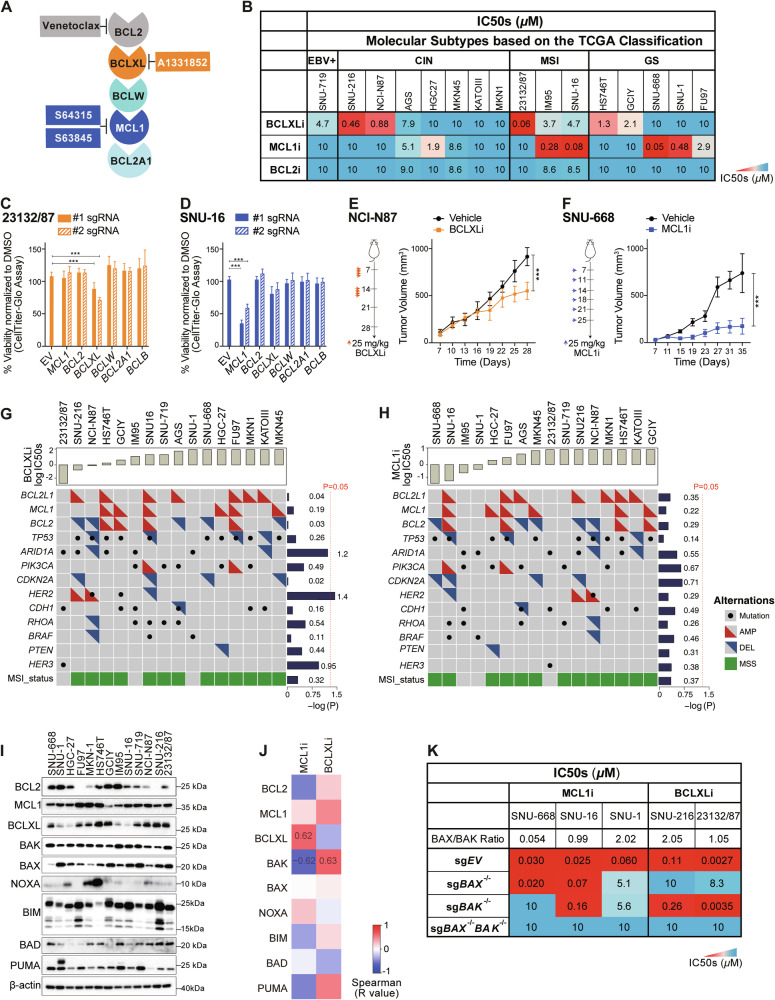
A subset of human GC cell lines is susceptible to single inhibition of BCLXL or MCL1. **A** Summary of the BH3-mimetic compounds used in this study. **B** Sensitivity of human GC cell lines to BH3-mimetic compounds. The sensitivity of 16 GC cell lines representing the four TCGA molecular subtypes to the indicated BH3-mimetics was determined after culturing in 0–10 μM of the drugs for 24 h. A discrete heat map representation of the mean IC50s was shown. **C** Genetic deleting *BCLXL* induces cell death in 23132/87 cells. The viability of 23132/87 cells 72 h after addition of doxocycline (DOX) to induce the expression of sgRNAs to target *BCL2*, *BCLXL*, *BCLW*, *MCL1*, *BCL2A1* or *BCLB* was determined with CellTiter-Glo assays. 2 sgRNAs were tested for each gene. Unpaired Student’s t-test was used for statistical significance. **D** SNU-16 cells lose their viability when *MCL1* is genetically ablated. Similar experiments to those in (**C**) were performed in SNU-16 cells. Anti-tumor activity of BCLXLi and MCL1i in vivo. BALB/c nude mice were inoculated s.c. with NCI-N87 (**E**) or SNU-668 (**F**) cells and treatment commenced 1 week later with BCLXLi (p.o.) or MCL1i (i.v.) as indicated. Tumor sizes were monitored every 3 days. Data shown represents the mean tumor volumes ± SD of 5 mice (**E**) and 7 mice (**F**) in each group. Statistical significance was calculated using the Two-way ANOVA analysis. **G**, **H** Correlation analysis of BCLXLi or MCL1i sensitivity and recurrent poor-risk genetic alterations in GC. All GC lines used in the study were ranked by increasing IC50s to BCLXLi (**G**) or MCL1i (**H**) treatment. The recurrent genetic alterations associated with poor prognosis in these cells were derived from the TCGA PanCancer Atlas study using the cBioPortal. Wilcoxon rank sum test was performed to determine the correlation between drug sensitivity and different genetic alterations. **I** Expression of BCL2 family proteins in the panel GC lines studied. **J** Spearman’s correlation analysis of BCL2 family protein expression and response to MCL1i and BCLXLi treatments. Spearman’s coefficient values with *P* value < 0.05 were shown in the plot. **K** Heatmap representation showing the impact of deleting *BAX*, *BAK,* or both on MCL1i and BCLXLi sensitivity. The mean IC50s ± SD of GC cell lines expressing sgRNAs targeting *BAX*, *BAK* or both to the indicated BH3-mimetic drugs were shown. Cell viability was determined using the CellTiter-Glo assay; data in (**B–D**) and (**K**) represent the means ± SD of ≥3 independent experiments; blots in (**I**) are representatives of 2 independent experiments. P values < 0.05 were considered significant. *P < 0.05, **P < 0.01, ***P < 0.001.

We and others have previously shown that only a subset of human GC cell lines is susceptible to BCLXL inhibition [12, 13], indicating that other pro-survival BCL2 proteins may play a role in maintaining GC cell survival. To investigate this, we assembled a larger panel of GC cell lines (n = 16) representing the four TCGA molecular subtypes of gastric cancer, including the Epstein-Barr Virus subtype (EBV+), microsatellite instability subtype (MSI), chromosomal instability subtype (CIN) and genomically stable subtype (GS) [28]. We firstly assessed their response to the well-characterized inhibitors of these proteins, including venetoclax to target BCL2 (referred to as BCL2i hereafter) [5], A1331852 to target BCLXL (referred to as BCLXLi hereafter) [29], and S63845 (in vitro assays) or S64315 (in vivo assays) to target MCL1 (both referred to as MCL1i hereafter) [30, 31] (Fig. 1A).

In line with our prior study [12], half GC lines tested (8/16) displayed varying degrees of susceptibility to BCLXL inhibition (IC50 < 5 µM), with 2 of them being highly sensitive (IC50 < 0.5 µM) [12]. Notably, 6 lines were also susceptible to MCL1 inhibition (IC50 < 5 µM), with 4 of them being highly sensitive (IC50 < 0.5 µM). However, none of them responded to BCL2i (Fig. 1B). Importantly, in addition to the MSI subtype, GC lines from the GS subtype, which typically benefit least from current therapies [32], responded to either BCLXL or MCL1 inhibitor. These results suggest that these GC subtypes could be promising candidates for BCLXL- or MCL1-targeting therapy. The effect of BCLXLi or MCL1i was confirmed to act through apoptosis, as loss of the downstream pro-apoptotic effectors BAX and BAK completely abolished the killing (Fig. S1A and S1B).

To validate the role of these proteins, we further employed CRISPR/Cas9 to disrupt the expression of all pro-survival BCL2 family genes individually in 23132/87 (BCLXLi-sensitive), SNU-16 and SNU-1 (MCL1i-sensitive) cells. Consistently, deleting *BCLXL* in 23132/87 cells or *MCL1* in SNU-16 and SNU-1 cells led to significant reduction in cell viability (Figs. 1C, D and S1C). In contrast, selective depletion of *BCLW*, *BCL2A1* or *BCLB* had no effect (Figs. 1C, D and S1C). Moreover, administration of BCLXLi or MCL1i significantly suppressed the growth of NCI-N87 or SNU-668 xenografts in vivo (Fig. 1E, F).

We next examined whether the responses to BCLXLi/MCL1i can be predicted by specific genetic feature(s). Interestingly, the effect of BCLXLi and MCL1i was independent of most recurrent high-risk genetic alterations in GC, except that GC lines with *HER2* amplification exhibited increased sensitivity to BCLXLi (p < 0.05) (Fig. 1G). Consistent with our previous studies [12], there is no significant correlation between *BCL2L1* amplification and BCLXLi sensitivity (Figs. 1G and S1D), nor between *MCL1* amplification and MCL1i sensitivity (Figs. 1H and S1E). The expression levels of BCL2 family proteins were reported to predict the response to BH3-mimetic drugs in some studies [30, 33, 34]. Consistently, we found a notable correlation between MCL1i sensitivity and BCLXL protein levels, with cells expressing lower levels of BCLXL being more sensitive [30, 34] (Figs. 1I, J, S1F, S1K). Although it was not statistically significant, there was a trend suggesting that cells with higher BCLXL protein expression were more susceptible to BCLXLi (Fig. S1G). Accordingly, we found that the expression levels of BCLXL protein cannot be predicted reliably by its gene amplification [12, 35, 36] (Fig. S1H). These results suggest that BCLXL protein levels, compared to *BCL2L1* CNVs, serve as a better biomarker in predicting the sensitivity of GC cells to MCL1 and BCLXL inhibitors.

Whilst the proapoptotic effector BAK is primarily guarded by MCL1 and BCLXL, divergent roles of BAX and BAK in mediating the killing of MCL1i have been reported [37–39]. Intriguingly, our correlation analysis indicated that while BAK expression is associated with increased sensitivity to MCL1i, it surprisingly predicts resistance to BCLXLi treatment (Figs. 1J, S1I and S1J). To further investigate this, we generated isogenic GC cell lines devoid of *BAX*, B*AK*, or both using CRISPR/Cas9 (Fig. S1K). Although the loss of *BAK*, not *BAX*, resulted in significant resistance to S63845 in SNU-668 with low BAX/BAK ratio (BAX/BAK = 0.054), both effectors exhibited roughly equivalent roles in SNU-16 (BAX/BAK = 0.99) and SNU-1 cells (BAX/BAK = 2.02) (Fig. 1K). Interestingly, BAX, but not BAK, was identified as the key mediator of BCLXLi in both SNU-216 (BAX/BAK = 2.05) and 23132/87 (BAX/BAK = 1.05) cells (Fig. 1K). These results suggest that the contribution of BAX/BAK in mediating the killing effect of MCL1i or BCLXLi cannot be simply accounted for by their relative expression but is influenced by other cellular factors that affect the function of BAX/BAK.

### Human GC cells are primed to undergo apoptosis and this is inhibited by both BCLXL and MCL1

**Fig. 2:**
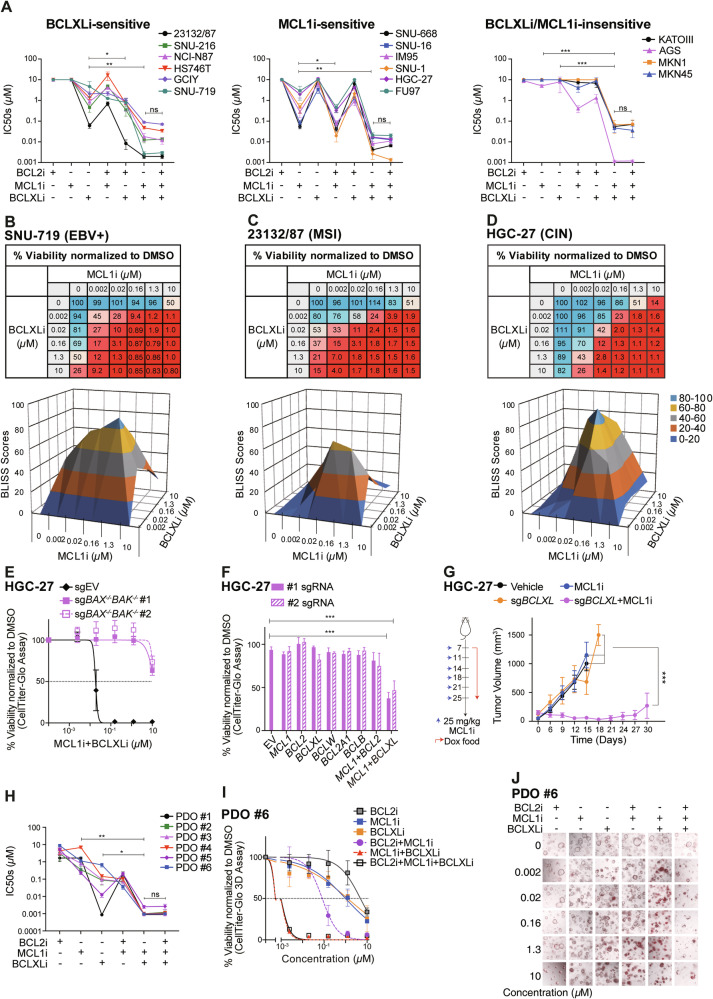
Dual inhibition of BCLXL and MCL1 act synergistically to kill both GC cell lines and patient-derived organoids. **A** Sensitivity of GC cell lines to different combinations of BH3-mimetic drugs. Similar experiments to those in Fig. 1B were performed with different combinations of BH3-mimetic drugs in equimolar concentrations (1:1 or 1:1:1). The responses of BCLXLi-sensitive, MCL1i-sensitive or MCL1i/BCLXLi-insensitive GC lines to the indicated treatments were summarized. Paired Student’s t-test was used to determine statistical significance. Synergistic killing by dual inhibition of BCLXL and MCL1. The viability of SNU-719 (**B**), 23132/87 (**C**) and HGC-27 (**D**) cells 24 h after treatment with increasing concentrations of indicated BH3-mimetic drugs was determined. BLISS scores were calculated and BLISS values > 0.0 indicate synergy between the two drugs at indicated concentrations. **E** BAX/BAK dependent killing of combining BCLXLi and MCL1i. The viability of WT and BAX/BAK deficient subclones of HGC-27 cells 24 h after treatment with the combination of BCLXLi and MCL1i (0–10 μM, 1:1) was determined. **F** Simultaneous deletion of BCLXL and MCL1 induces cell death in HGC-27 cells. Similar experiments to those in Fig. 1C, D were performed in HGC-27 cells. **G** Loss of BCLXL enhances MCL1 inhibition in vivo. HGC-27 cells inducibly expressing sgBCLXL or the sgRNA empty vector were subcutaneously inoculated into the BALB/c nude mice and treatment commenced 1 week later with doxycycline food alone or together with MCL1i. Tumor sizes were monitored every 3 days. Data shown represents the mean tumor volumes ±SD of 6 mice in each group. **H** Sensitivity of patient-derived organoids to the BH3-mimetic treatment. Established tumoroids were dissociated and seeded as single cells, grown into organoids over 7–9 days and treated with the indicated BH3-mimetic drugs, alone or in equimolar combinations (1:1 or 1:1:1). Cell viability was determined 24 h later using CellTitre-Glo 3D assays. Paired Student’s t-test was used to determine statistical significance. **I** Representative dose response curves of organoid #6 shown in (**H**). **J** The response of organoid #6 to different BH3-mimetic treatments was determined by PI (red) staining. Representative images at 72 h post-treatment were shown. Cell viability was determined using the CellTiter Glo or CellTiter Glo 3D assay; data in (**A–F**), (**H**) and (**I**) represent the means ± SD of ≥3 independent experiments; data in (**J**) are representatives of 2 independent experiments.

The non-targeted pro-survival BCL2 family members can limit the full action of BH3-mimetic compounds to trigger apoptosis [40]. We therefore examined whether a combination of them could enhance the killing of GC cells. Unlike the defective apoptotic machinery reported in some AML [41] and colorectal cancer subsets [42], all tested GC lines were effectively killed by the triple combination of BCL2, BCLXL, and MCL1 inhibitors. Strikingly, dual inhibition of BCLXL and MCL1 killed all GC lines as efficiently as the triple combination, with negligible effect from adding a BCL2 inhibitor (Figs. 2A, S2A and S2B). Even in cell lines susceptible to single inhibition of BCLXL or MCL1, dual inhibition could markedly accelerate the rates of apoptosis and achieve enhanced cytotoxicity against GC cells (Figs. 2A, S2A–S2C). BLISS analysis further confirmed the strong synergy of combining BCLXL and MCL1 inhibitors across multiple GC lines (Figs. 2B–D, S2D and S2E). The effects of these combinations were confirmed to occur through apoptosis (Figs. 2E and S2F). The role of BCLXL and MCL1 in maintaining GC cell survival was further confirmed genetically in HGC-27 cells (Fig. 2F).

We next tested whether co-targeting them can enhance the killing of GC cells in vivo. Due to the lethality of direct combination of BCLXL and MCL1 inhibitors in mice [43], we employed an alternative approach using doxycycline-inducible sgRNA to specifically disrupt *BCLXL* expression and combined it with the MCL1 inhibitor. As expected, although the single inhibition of BCLXL (by sg*BCLXL*) or MCL1 (by MCL1 inhibitor) did not affect the growth of HGC-27 xenografts, co-targeting BCLXL and MCL1 led to a substantial suppression of tumor growth in vivo (Fig. 2G).

### Human gastric cancer derived organoids rely predominantly on BCLXL for survival, and to a lesser extent, on MCL1

Our data from GC cell lines suggested that BCLXL and MCL1 are the key pro-survival factors to target to kill GC cells. We next tested whether it also holds true in a more clinically relevant model using patient-derived tumor organoids (PDOs). PDOs were generated from 6 GC patients. Remarkably, 5/6 PDOs were readily killed by BCLXL inhibition alone (IC50 < 0.5 µM), with one tumoroid (PDO #6) exhibiting moderate sensitivity (IC50: 0.66 µM) (Fig. 2H, I). 2/6 tumoroids can also be readily killed by MCL1i alone (IC50 < 0.5 µM). Consistent with GC cell lines, none of them responded to BCL2i. We also examined the effect of different combinations in killing these tumoroids. PDO #1 appeared to rely predominantly on BCLXL for survival, with no further increase in killing by adding MCL1i or BCL2i. Of note, dual inhibition of BCLXL and MCL1 exhibited markedly enhanced killing in the remaining PDOs. Although adding BCL2i could also increase the killing of MCL1i in some PDOs (#1-#4, #6), it was much less effective compared to the MCL1i/BCLXLi combination. Similar observations were made using an orthogonal approach to monitor the viability of PDOs via PI staining followed by imaging (Fig. 2J). Collectively, our results in PDOs recapitulated our observations in GC cell lines and highlighted the important role of BCLXL and MCL1 in maintaining GC cell survival.

### Anti-mitotic chemotherapies induce MCL1 degradation and synergize with BCLXL inhibitor to kill GC cells

Our data from both GC cell lines and PDOs revealed the potent synergistic activity of co-targeting BCLXL and MCL1 in promoting GC apoptosis. However, direct combination of BCLXL and MCL1 inhibitors is not possible due to the on-target toxicity associated with direct BCLXL and MCL1 inhibitors in vivo. Compared to the unsolved cardiotoxicity associated with direct MCL1 inhibitors, it has been reported that low levels of thrombocytes caused by BCLXL inhibition can potentially be managed by carefully adjusting the dose of the drug [44, 45]. Thus, finding combinations of agents that indirectly target MCL1 and enable the reduction of doses of BCLXL inhibitors while maintaining their anti-neoplastic effects, is potentially a practicable approach for the further development of these drug classes.

**Fig. 3:**
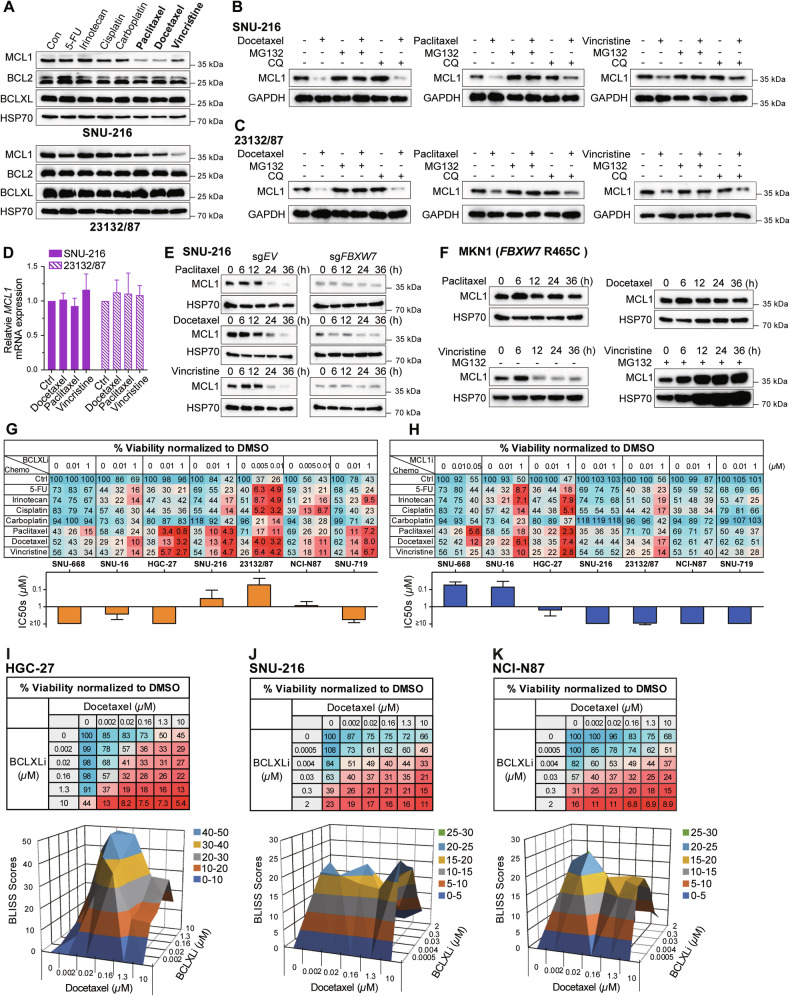
Anti-mitotic chemotherapies induce MCL1 degradation and synergize with BCLXL inhibitor to kill GC cells. **A** Effect of different chemotherapies on MCL1, BCLXL and BCL2 expression. SNU-216 and 23132/87 cells were treated with DMSO (Con) or 10 μM of the indicated chemotherapies for 24 h and the protein levels of MCL1, BCL2, BCLXL were determined by Western blotting. Ubiquitin-mediated protein degradation of MCL1 upon treatment with anti-mitotic drugs. SNU-216 (**B**) and 23132/87 cells (**C**) were treated with anti-mitotic drugs alone (10 μM) or together with inhibitors of proteasome (MG132, 5 μM) or lysosome (CQ, 5 μM) for 24 h and the protein levels of MCL1 were determined by Western blotting. **D** Effect of anti-mitotic drugs on *MCL1* mRNA level. SNU-216 and 23132/87 cells were treated with indicated drugs (10 μM) for 24 h and the mRNA levels of *MCL1* were determined by RT-qPCR. **E** Role of FBXW7 in mediating the degradation of MCL1 by anti-mitotic drugs. SNU-216 cells transduced with sgRNA targeting *FBXW7* or the sgRNA empty vector were treated with anti-mitotic drugs (10 μM) for the indicated time periods. The protein levels of MCL1 were determined by Western blotting. **F** Effect of anti-mitotic drugs on MCL1 degradation in *FBXW7*-mutant cells. *FBXW7*-mutant MKN1 (R465C) cells were treated with anti-mitotic drugs alone (10 μM) or together with MG132 (5 μM) for the indicated time points and the protein levels of MCL1 were determined by Western blotting. **G**, **H** In vitro activity of BCLXLi or MCL1i in combination with different chemotherapies. The responses of MCL1i-sensitive (SNU-668, SNU-16, HGC-27), BCLXLi-sensitive (SNU-216, 23132/87, NCI-N87) or the MCL1i/BCLxLi-insensitive (SNU-719) GC lines to the indicated concentrations of BCLXLi/MCL1i alone or in combination with chemotherapies (10 μM) were determined 72 h post-treatment. **I–K** Strong synergy between docetaxel and BCLXL inhibition. The viability of HGC-27 cells was determined 24 h after treatment with increasing concentrations of docetaxel and BCLXLi, followed by BLISS score analysis. Cell viability was determined using the CellTiter-Glo assay; data in (**D**) and (**G–K**) represent the means ± SD of ≥3 independent experiments; blots in (**A–C**), (**E**) and (**F**) are representatives of 2 independent experiments.

We next examined the effect of seven commonly used chemotherapy drugs in GC treatment on the expression levels of BCL2 family proteins. Notably, all three anti-mitotic drugs induced significant reduction of MCL1, but not BCL2 or BCLXL protein levels, in both SNU-216 and 23132/87 cells (Figs. 3A and S3A). We further investigated how these anti-mitotic drugs reduced the protein levels of MCL1. Interestingly, the co-treatment with MG132 to block the ubiquitin-proteasome pathway effectively prevented the reduction of MCL1 protein levels (Figs. 3B, 3C and S3A). In contrast, the co-treatment with chloroquine (CQ) to block the lysosomal pathway or the pan-caspase inhibitor Q-VD-Oph had no effect. The latter excluded the possibility of MCL1 cleavage caused by chemotherapy-induced caspase activation [46] (Figs. 3B, 3C and S3B). Moreover, there was no change in *MCL1* mRNA levels (Fig. 3D). These results indicated that these anti-mitotic drugs induced MCL1 degradation via the ubiquitin-proteasome pathway.

Several E3 ligases, including FBXW7, MARCH5, APC/C^Cdc20^ and MULE, have been reported to mediate MCL1 degradation upon anti-mitotic treatment [47, 48]. *FBXW7* deletions or loss-of-function mutations are associated with poor prognosis and therapy resistance in GC [49]. We thus first examined the role of FBXW7 in this context and found that deleting *FBXW7* successfully blocked MCL1 degradation triggered by these drugs (Figs. 3E and S3C). R465C/H is the most prevalent *FBXW7* mutation among GC patients (Figure S3D). Interestingly, MCL1 persisted in *FBXW7*-mutant MKN1 (R465C) cells following treatment with docetaxel and paclitaxel, but not with vincristine (Fig. 3F). Nevertheless, adding MG132 also blocked MCL1 degradation upon vincristine treatment, indicating that the decrease of MCL1 protein levels in this context still occurs through the ubiquitin-proteasome pathway but is mediated by other E3 ligase(s). Based on these results, we proposed that combining anti-mitotic chemotherapies (targeting MCL1 indirectly) with a BCLXL inhibitor is prone to achieve enhanced killing of GC cells.

We therefore evaluated the effect of combining these chemotherapy drugs tested above with BH3-mimetic drugs in a panel of GC lines with distinct reliance on these pro-survival BCL2 proteins. Strikingly, inhibiting BCLXL markedly increased the cytotoxicity of anti-mitotic drugs in all tested GC lines, regardless of their reliance on different pro-survival BCL2 proteins (Fig. 3G). Although inhibiting MCL1 also increased the activity of anti-mitotic drugs in MCL1-reliant GC cell lines, this effect was mainly observed at higher dose of the drug (1 µM in SNU-16 and HGC-27), which showed limited effect in the remaining cell lines (Fig. 3H). Inhibiting BCL2 had no role on enhancing the effect of these drugs (Fig. S3E). Although an enhanced response to 5-FU, irinotecan and cisplatin was also observed by inhibiting BCLXL or MCL1 in some cases, none of these effects were as profound as those observed when combining anti-mitotic agents with the BCLXL inhibitor. BLISS analysis further confirmed the synergistic effect of combining docetaxel and BCLXLi (Fig. 3I–K). Importantly, the loss of BAX and BAK efficiently blocked cell death induced by the combinations of anti-mitotic drugs and BCLXLi determined by PI staining (Fig. S3F and S3G).

### BCLXL inhibitor markedly augments the anti-tumor effect of docetaxel in PDOs and xenograft models

**Fig. 4:**
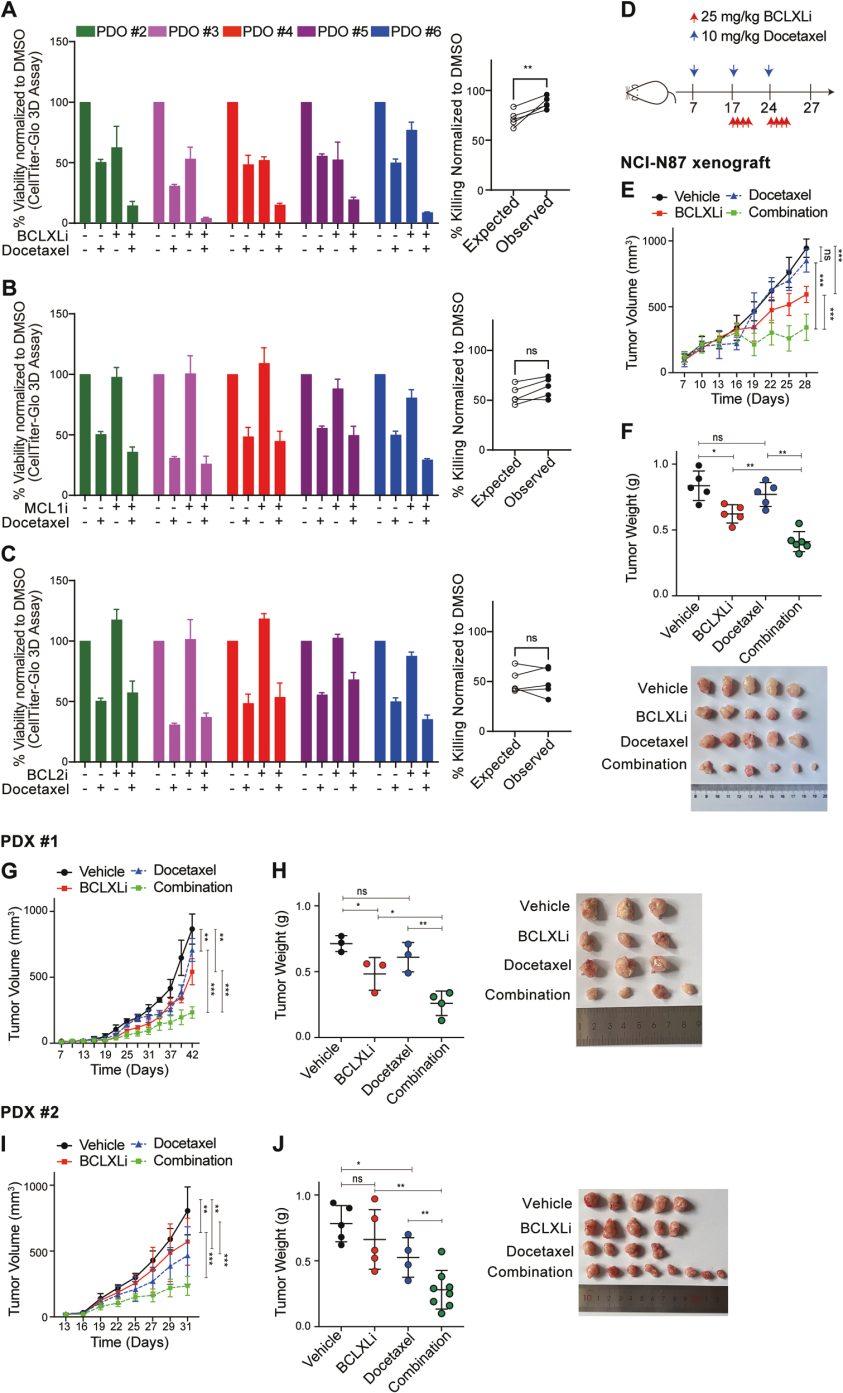
Inhibition of BCLXL markedly enhances the anti-tumor activity of docetaxel in PDOs and xenograft models. **A–C** In vitro activity of BH3-mimetic drugs in combination with docetaxel in PDOs. Left panel: PDOs #2–#6 in Fig. 2H were treated with vehicle, 500 nM docetaxel, BH3-mimetic drug, or both. The concentrations of BCLXLi, MCL1i and BCL2i: PDO #2: 20 nM, PDO #3: 10 nM, PDO #4: 100 nM, PDO #5: 10 nM, PDO #6: 100 nM. Cell viability was determined 72 h later using CellTitre-Glo 3D assays and the mean IC50s ± SD of 3 independent experiments are shown. Right panel: Synergistic activity of different combinations. The expected versus observed effects of BH3-mimetic drugs in combination with docetaxel were calculated using the BLISS model. Unpaired Student’s t-test was used to determine statistical significance. **D** Schedule and doses of drug treatment for in vivo studies. **E** Inhibition of BCLXL markedly enhanced the anti-tumor activity of docetaxel in vivo. NCI-N87 xenografts were treated as indicated and tumor growth was summarized. Data shown represent the mean tumor volumes ± SD of 5–6 mice in each group. Two-way ANOVA was used for statistical significance. **F** Images (top) and quantification of tumor weights (bottom) in the NCI-N87 xenografts. Unpaired Student’s t-test was used for statistical significance. **G**–**J** Similar experiments to those in (**E**, **F**) were performed in two PDX xenografts.

We next tested whether the synergistic activity of anti-mitotic agents and BCLXL inhibition also occurs in primary GC cells. The combination of a low dose of BCLXLi and docetaxel led to a markedly improved cytotoxicity against tumoroids. Notably, the combination of docetaxel and BCLXLi, but not MCL1i and BCL2i, exhibited synergy (Fig. 4A–C and S4A–S4D). Importantly, combining BCLXLi markedly enhanced the anti-tumor efficacy of docetaxel in the NCI-N87 and two PDX xenograft models (Fig. 4D–J). Compared to single treatments, combining docetaxel and BCLXLi significantly delayed tumor progression, reduced tumor volume and weight in all three xenograft models (Fig. 4E–J), Moreover, the combination therapy appeared to be well tolerated while the mice maintained normal body weight during therapy (Fig. S4E and S4F).

### HER2-targeting drugs suppress *MCL1* transcription and synergize with BCLXL inhibitor to kill *HER2*-amplified GC cells

**Fig. 5:**
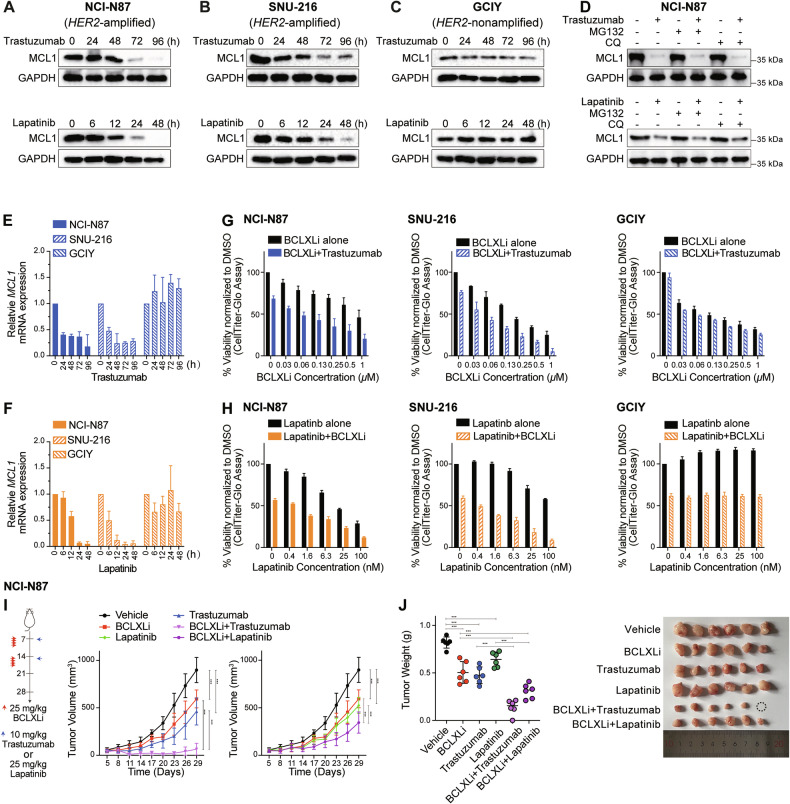
HER2-targeting drugs suppress *MCL1* transcription and synergize with BCLXL inhibitor to kill *HER2*-amplified GC cells. **A–C** Effect of HER2-targeting drugs on MCL1 protein levels. NCI-N87 (HER2-amplifed), SNU-216 (HER2-amplified) and GCIY (HER2-nonamplified) were treated with trastuzumab (100 ng/ml) or lapatinib (200 nM) for the indicated time periods. The protein levels of MCL1 were determined by Western blotting. **D** Blocking ubiquitin- or lysosome-mediated protein degradation failed to reverse the decrease in MCL1 protein levels. NCI-N87 cells were treated with trastuzumab (100 ng/ml), lapatinib (200 nM) alone or together with inhibitors of proteasome (MG132, 5 μM) or lysosome (chloroquine, CQ, 5 μM) for 72 h or 48 h respectively. The protein levels of MCL1 were determined by Western blotting. **E**, **F** Effect of trastuzumab and lapatinib on *MCL1* mRNA levels. NCI-N87, SNU-216 and GCIY were treated with trastuzumab (100 ng/ml) or lapatinib (200 nM) for the indicated time periods. The mRNA levels of *MCL1* were determined by RT-qPCR. **G** In vitro activity of combining BCLXLi and trastuzumab. The cell viability of NCI-N87, SNU-216 or GCIY 72 h after treatment with 100 ng/ml trastuzumab alone or in combination with indicated concentrations of BCLXL inhibitor was determined. **H** In vitro activity of combining BCLXLi and lapatinib. The cell viability of NCI-N87, SNU-216, or GCIY 48 h after treatment with lapatinib alone (0–100 nM) or in combination with BCLXL inhibitor was determined. The concentrations of BCLXLi used: 10 nM, 20 nM, and 1 µM for NCI-N87, SNU-216, and GCIY, respectively. **I** Inhibition of BCLXL markedly enhanced the anti-tumor activity of HER2-targeting drugs in vivo. NCI-N87 xenografts were treated as indicated and tumor growth was summarized. Data shown represents the mean tumor volumes ± SD of 5–6 mice in each group. Two-way ANOVA was used to determine statistical significance. **J** Images (right) and quantification of tumor weights (left) in the NCI-N87 xenografts. Unpaired Student’s t-test was used to determine statistical significance. Cell viability was determined using the CellTiter-Glo assay; blots in (**A–D**) are representatives of 2 independent experiments; data in (**E**–**H**) represent the means ± SD of ≥3 independent experiments.

Our early analysis revealed that GC cell lines with *HER2* amplification exhibited increased sensitivity to BCLXLi (Fig. 1G). Although HER2-targeting drugs (e.g., trastuzumab and lapatinib) have been reported to regulate MCL1 activity via distinct mechanisms (transcriptionally or translationally) in other cancer types [50–52], their effects in GC cells have not been well studied. Interestingly, both trastuzumab and lapatinib induced significant down-regulation of MCL1 protein levels in *HER2*-amplified NCI-N87 (Fig. 5A) and SNU-216 (Fig. 5B), but not in *HER2*-nonamplified GCIY cells (Fig. 5C). However, co-treatment with MG132 or CQ failed to reverse the reduction of MCL1 protein levels (Figs. 5D and S5A), indicating that trastuzumab/lapatinib regulates MCL1 expression at the transcriptional level. Accordingly, both drugs induced significant reduction of *MCL1* mRNA in *HER2*-amplified cells (Fig. 5E, F). These results provide a rationale for combining BCLXL inhibitor with HER2-targeting drugs to potentially achieve enhanced anti-tumor activity in *HER2*-amplified GC.

We firstly evaluated the effect of combining trastuzumab/lapatinib with BCLXLi in vitro. As expected, inhibiting BCLXL augmented the activity of trastuzumab/lapatinib in both NCI-N87 and SNU-216, but not in GCIY cells (Fig. 5G, H). Importantly, the loss of *BAX* and *BAK* efficiently blocked the effect induced by these treatments (Fig. S5B and S5C). Although MCL1 inhibition was reported to synergize with HER2-targeting drugs in breast cancer [50], the addition of MCL1i or BCL2i (up to 2 μM) failed to enhance the activity of trastuzumab in *HER2*-ampfiled GC cells (Fig. S5D and S5E). Moreover, compared to single-drug treatments, combining BCLXLi with trastuzumab/lapatinib markedly enhanced the suppression of tumor progression and reduced tumor volume and weight in NCI-N87 xenografts (Fig. 5I, J). The combination of BCLXLi with HER2-targeting drugs appeared to be well tolerated with mice maintaining normal body weight during therapy (Fig. S5F).

### STAT3 cooperates with SRF to mediate the transcriptional response of *MCL1* to HER2-targeting drugs

**Fig. 6:**
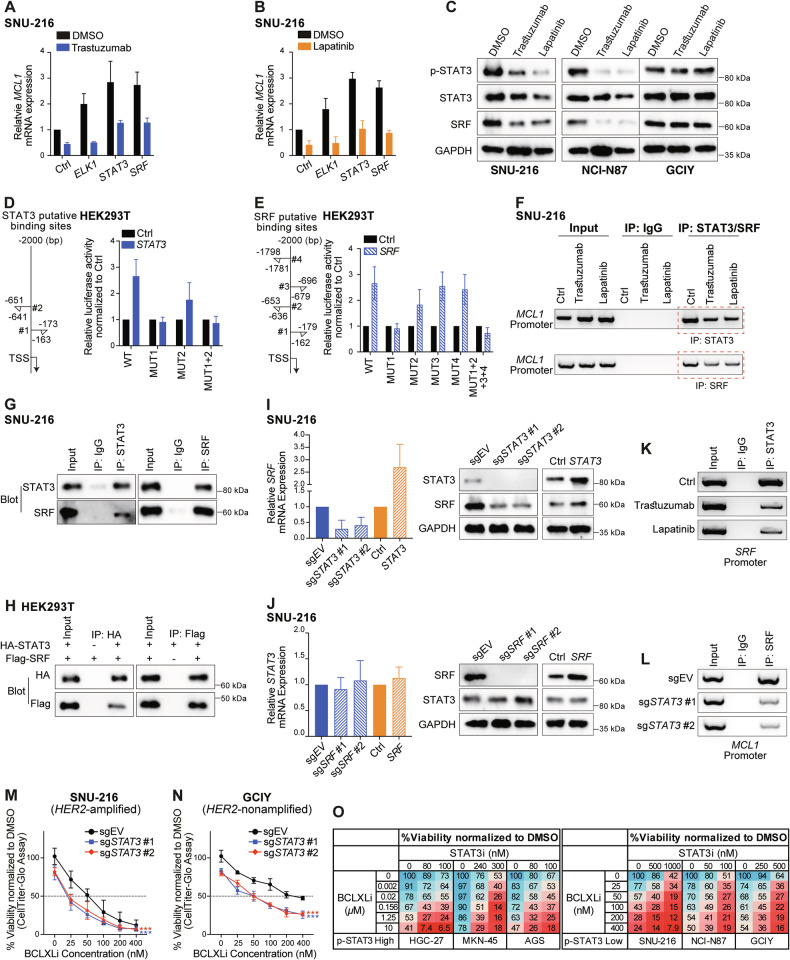
STAT3 and SRF cooperate to mediate the transcriptional response of *MCL1* exerted by HER2-targeting drug treatment. **A**, **B** Role of ELK1, STAT3 and SRF in regulating the transcriptional activity of *MCL1* under basal conditions and in response to HER2-targeting drugs. SNU-216 cells expressing empty vector or the indicated transcriptional factors were left untreated or treated with 100 ng/ml trastuzumab (**A**) or 200 nM lapatinib (**B**) for 72 h or 48 h, respectively. The levels of *MCL1* mRNA were determined using RT-qPCR. **C** Effect of HER2-targeting drugs on STAT3 and SRF expression. SNU-216, NCI-N87 and GCIY were treated with 100 ng/ml trastuzumab or 200 nM lapatinib for 72 h or 48 h, respectively. The protein levels of p-STAT3, total STAT3, and SRF were determined by Western blotting. **D**, **E** Dual luciferase reporter assays to identify the binding site(s) of STAT3 and SRF in the *MCL1* promoter. Left: diagram depicting the putative binding sites of STAT3 (**D**) or SRF (**E**) in the *MCL1* promoter predicted by the JASPAR database. Right: Dual luciferase reporter assays conducted in HEK293T. **F** Reduced binding of STATS and SRF in the *MCL1* promoter upon HER2-targeting drug treatments. SNU-216 cells were left untreated or treated with 100 ng/ml trastuzumab or 200 nM lapatinib for 72 h or 48 h, respectively. ChIP was conducted using specific antibodies against STAT3, SRF, and an IgG isotype control. **G** Co-IP analysis of the interaction of endogenous STAT3 and SRF in SNU-216 cells. **H** Direct interaction of exogenous STAT3 and SRF. HEK293T cells were transiently transfected with HA-tagged STAT3, Flag-tagged SRF, either individually or both. Equivalent lysates were immunoprecipitated with anti-HA or anti-Flag antibody and immunoblotted with the indicated antibodies. **I** Effect of overexpressing or deleting *STAT3* on SRF expression. The mRNA (left) and protein (right) levels of SRF in SNU-216 cells with STAT3 overexpression or depletion were determined. **J** Similar experiments to those in panel I were conducted to detect the effect of manipulating *SRF* on STAT3 expression in SNU-216 cells. **K** Reduced binding of STAT3 in the *SRF* promoter upon HER2-targeting drug treatments. Similar experiments to those in (**F**) were conducted. **L** Depletion of *STAT3* attenuated the binding of SRF in the *MCL1* promoter. Similar experiments to those in (**F**) were performed in SNU-216 cells engineered to express sg*STAT3*. ChIP was conducted at 72 h after addition of DOX to induce the deletion of *STAT3*. 2 sgRNAs were tested. **M**, **N** Deleting *STAT3* enhanced the sensitivity of GC lines to BCLXL inhibition. SNU-216 (**M**) and GCIY (**N**) cells inducibly expressing sg*STAT3* or the sgRNA empty vector were treated with DOX alone, or in combination with indicated concentrations of BCLXLi. Cell viability was determined 48 h later. Two-way ANOVA was used for statistical significance. **O** STAT3 inhibitor synergized with BCLXLi in GC cell lines, regardless of their p-STAT3 levels. GC cell lines with high p-STAT3 levels (HGC-27, MKN45 and AGS) and the ones with low p-STAT3 levels (SNU-216, NCI-N87, GCIY) were treated with indicated concentrations of BCLXLi and STAT3 inhibitor and cell viability was determined 48 h later. Data in (**A**), (**B**), (**D**), (**E**), (**I**, **left**), (**J**, **left**), (**M**–**O**) represent the means ± SD of ≥3 independent experiments; data in panel (**C**), (**F**–**H**), (**I**, **right**), (**J**, **right**), (**K**) and (**L**) are representatives of 2 independent experiments.

We next sought to identify the transcriptional factor(s) responsible for regulating *MCL1* transcription exerted by HER2-targeting drugs in these GC cells. ELK1, but not SRF or STAT3, was reported to regulate both the basal and epidermal growth factor induced *MCL1* transcription in breast cancer cells [53]. Although overexpression of ELK1 upregulated the basal transcription of *MCL1* mRNA, it failed to reverse the reduction of *MCL1* mRNA expression upon trastuzumab/lapatinib treatment (Figs. 6A, B and S6A). NF-κB was also reported to regulate *MCL1* transcription downstream of the MEK/ERK signaling pathway upon sorafenib treatment in HCT116 cells [54]; however, it did not have a role in GC cells (Fig. S6B–S6D). Interestingly, we found that overexpression of STAT3 and SRF not only increased the basal transcriptional activity of *MCL1* but also rescued the reduction of *MCL1* mRNA expression upon trastuzumab/lapatinib treatment (Figs. 6A, B and S6A). Accordingly, we observed decreased p-STAT3 and SRF levels upon trastuzumab/lapatinib treatment in SNU-216 and NCI-N87, but not in GCIY cells (Fig. 6C). Intriguingly, although depletion of *ELK1*, *STAT3* or *SRF* reduced the basal transcriptional activity of *MCL1* as expected, depletion of *STAT3* or *SRF*, but not *ELK1*, also abolished the transcriptional suppression of *MCL1* upon trastuzumab/lapatinib treatment (Fig. S6B–S6D). This indicates that targeting STAT3 or the JAK/STAT3 pathway may antagonize with HER2-targeting drugs, which might explain why the combination of the JAK inhibitor ruxolitinib with trastuzumab failed to improve the outcomes in trastuzumab-resistant HER2-positive metastatic breast cancer [55]. SRF generally associates with the ERK-regulated ternary complex factors (ELK1, ELK3, or ELK4) to regulate cell proliferation and growth [56]. However, neither ELK3 nor ELK4 had a role in these processes (Fig. S6E and S6F). Collectively, these results demonstrate that STAT3 and SRF mediate the transcriptional response of MCL1 to HER2-targeting drug treatment, with SRF’s effect being independent of the typical ternary complex factors.

We next investigated the regulatory elements required for STAT3/SRF to exert the impact on *MCL1* transcription. Consistent with the data in SNU-216 (Fig. 6A, B), overexpression of STAT3 or SRF elevated the luciferase activity of reporter plasmids containing wildtype *MCL1* promoter in HEK293T cells. This was abolished when STAT3 #1 and SRF #1 binding sites were mutated in the *MCL1* promoter (Fig. 6D,E). The binding of STAT3 and SRF within the *MCL1* promoter was further confirmed by ChIP PCR analysis in SNU-216 cells, which was reduced upon trastuzumab/lapatinib treatment accordingly (Fig. 6F).

Since the binding sites for STAT3 and SRF overlap within the *MCL1* promoter, a direct interaction between these two factors may exist. To test this, a series of co-IP assays were conducted in SNU-216 cells, which showed that STAT3 and SRF physically interact (Fig. 6G). The direct interaction of STAT3 and SRF was further confirmed in HEK293T cells overexpressing exogenous HA-tagged STAT3 and Flag-tagged SRF (Fig. 6H). It is worth mentioning that SRF levels were also decreased upon trastuzumab/lapatinib treatment, indicating that SRF may act downstream of STAT3. Indeed, we found that STAT3 regulates *SRF* transcription by directly binding to the *SRF* promoter (Fig. 6I–K). Accordingly, the depletion of *STAT3* reduced the binding of SRF to the *MCL1* promoter (Fig. 6L). These results suggest that HER2-targeting drugs suppress STAT3 activity, and this, in turn, downregulates *SRF* transcription. Furthermore, STAT3 and SRF cooperate through their direct interaction to regulate *MCL1* transcription under both basal conditions and in response to trastuzumab/lapatinib treatment.

### Enhanced killing by co-targeting BCLXL and STAT3, extending beyond *HER2*-amplified GC cells

Given the role of STAT3 and SRF in mediating the transcription response of *MCL1* to HER2-targeting drugs in GC cells, we next explored whether targeting STAT3 or SRF also synergized with BCLXLi to kill *HER2*-amplified GC cells. Interestingly, markedly enhanced killing was achieved by co-treating SNU-216 cells with doxycycline (to induce deletion of *STAT3*) and BCLXLi (Figs. 6M and S6D). Although the effect was less pronounced, depletion of *SRF* in combination with BCLXLi also increased the killing of SNU-216 cells (Fig. S6G). Moreover, markedly enhanced killing was observed when GCIY cells were co-treated with doxycycline (to induce deletion of *STAT3*) and BCLXLi (Fig. 6N). Accordingly, reduced MCL1 protein expression was detected in GCIY cells with *STAT3* depletion (Fig. S6H).

BBI608 (Napabucasin) is a small molecule inhibitor of STAT3, which has advanced into phase III trials in GC [57]. Although p-STAT3 was reported to act as a predictive biomarker of BBI608’s clinical activity in cancer patients, it failed to predict the clinical activity in some studies [57, 58]. We next examined the levels of p-STAT3 (Fig. S6I) and selected the top 3 GC lines with the highest p-STAT3 expression and the bottom 3 GC lines with the least p-STAT3 expression to test their response to BBI608 alone and in combination with BCLXLi. Interestingly, no clear correlation between p-STAT3 levels and BBI608 response was detected (Fig. S6J). Nevertheless, combining BCLXLi markedly enhanced the killing of these GC cells by BBI608, regardless of their p-STAT3 levels (Fig. 6O). Collectively, our results demonstrated profound anti-tumor activity of co-targeting BCLXL and STAT3, extending beyond *HER2*-amplified GC cells.

## Discussion

Advanced GC remains a significant challenge in clinical practice. While the addiction of many solid cancer entities to pro-survival BCL2 proteins has been reported [44], their roles in GC have not been well investigated. Using both pharmacological and genetic approaches, we found that MCL1, in addition to BCLXL, plays a crucial role in GC cell survival, with a negligible role with other pro-survival BCL2 relatives. While targeting BCLXL or MCL1 exhibits some single-agent activity, their combined inhibition is sufficient to cause maximum killing in both GC cell lines and PDOs, regardless of their molecular subtypes. Our findings suggest that BCLXL and MCL1 are highly promising drug targets in GC, including high-risk cases.

Divergent roles of BAX and BAK in mediating the killing effect of BH3 mimetic drugs have been reported. Our previous study has reported that BAK expression serves as a positive marker for predicting sensitivity to the MCL1 inhibitor AM8621 [37]. Moreover, using a genome-wide CRISPR knockout screen, we found that deletion of *BAK*, but not *BAX*, led to resistance to S63845 in the SK-RB-3 breast cancer cell line [50]. On the other hand, BAX was also reported to play a primary role in apoptosis induced by S63845 across several hematopoietic malignancies, including multiple myeloma, Eµ-Myc, and diffuse large B-cell lymphoma cells [38, 39, 59]. The role of BAX and BAK in the action of BCLXL inhibitors is not clear. We found that, in most cases, the effect of S63845 in GC cells was mediated by both BAX and BAK. Intriguingly, BAX, rather than BAK, was identified as the primary mediator of BCLXLi. These studies suggest that the role of BAX/BAK in mediating the killing effect of these BH3-mimetic drugs is cell type- and disease-dependent and cannot be simply accounted for by their relative expression levels.

The development of BCLXL-targeting therapies for cancer therapy has been impeded due to the major side effects of thrombocytopenia caused by navitoclax [45]. In a recent phase I study, the combination of venetoclax with low-dose navitoclax and chemotherapy was reported to be well tolerated and demonstrated promising efficacy in patients with relapsed/refractory acute lymphoblastic leukemia or lymphoblastic lymphoma [60]. This indicates that low levels of thrombocytes can potentially be managed by carefully adjusting the dose of navitoclax. Moreover, efforts are underway to reduce the on-target toxicity of BCLXL targeting on platelets, including the use of a VHL-based proteolysis-targeting chimera (PROTAC) (e.g., DT2216) [61] and an antibody-drug conjugate (e.g., ABBV-155). These developments represent important steps toward safer and more effective BCLXL-targeting therapies in cancer treatment.

The therapeutic potential of MCL1 inhibitors, both as monotherapy and in combination with BCL2 inhibitors like venetoclax, is being explored in various hematologic malignancies. However, clinical development has been complicated by safety issues, notably cardiac toxicity, which led to the suspension of clinical trials with several MCL1 inhibitors. In addition to hematological cancers [6], some solid tumors, such as breast cancer, melanoma, and pancreatic ductal adenocarcinoma, were also reported to be reliant on MCL1 for survival [34]. Here, we demonstrated that GC cells with low BCLXL protein expression rely predominantly on MCL1 for survival. However, due to the unsolved cardiotoxicity of direct MCL1 inhibitors, identification of drugs that suppress MCL1 activity indirectly may be a feasible approach to target MCL1 at the current stage. Importantly, we identified three classes of drugs, including anti-mitotic drugs, HER2-targeting drugs as well as the STAT3 inhibitor, which down-regulate the activity of MCL1 via distinct mechanisms. These drugs synergize significantly with a BCLXL inhibitor and lead to enhanced anti-tumor activity with tolerable toxicity in preclinical GC models.

Several clinical trials evaluating navitoclax in combination with different chemotherapeutic drugs, including cisplatin, irinotecan, taxol, etc., in solid tumors are under way. However, our study, along with those of others, has reported that navitoclax acts as a much more potent inhibitor of BCL2 than BCL-XL [12, 62]. Our results in GC suggest that inhibiting BCL2 had no role on increasing the effect of these drugs and targeting BCLXL showed more synergistic activity with anti-mitotic drugs than other standard chemotherapeutic drugs. Moreover, our study, for the first time, identified the strong synergistic activity when combining BCLXL inhibitor with HER2-targeting drugs or the STAT3 inhibitor. Thus, it will be revealing to conduct clinical trials to evaluate the safety profile of selective BCLXL-targeting drugs and their compatibility with these drugs in combination therapies. The strong synergistic activity observed between these drugs may allow for reduced dosages, potentially minimizing their adverse effects.

Taken together, our results underscore the vulnerability of GC cells to the induction of apoptosis and identify BCLXL and MCL1 as the two critical factors in maintaining the survival of GC cells. We anticipate that targeting these proteins, either directly or through rational combination therapy, could significantly improve the prognosis for GC patients, particularly for those in advanced stages of the disease, provided that safety concerns are thoroughly addressed.

## Materials and methods

### Human samples

Fresh tumor tissues were collected from GC patients at surgery with informed consent. The study was performed with the approval of National Cancer Center/National Clinical Research Center for Cancer/Cancer Hospital (17-156/1412), and the Institute of Laboratory Animal Sciences (GJN22002), Chinese Academy of Medical Sciences and Peking Union Medical College. A summary of patient information is provided in Table S1.

### Mice

Female BALB/c Nude and NTG mice (6–8 weeks of age) were purchased from Sibeifu Biotechnology Co., Ltd (Beijing, China) and maintained in specific pathogen free animal research facility. Upon arrival, mice were allowed to acclimate for 2–3 days before experiment. The study was approved by the Institutional Animal Care and Use Committee of the Institute of Laboratory Animal Science, Chinese Academy of Medical Sciences and Peking Union Medical College (IACUC 21001).

### Cell lines and culture

Human GC cell lines, including SNU-719, SNU-216, NCI-N87, AGS, HGC-27, MKN45, KATOIII, MKN1, 23132/87, IM95, SNU-16, HS746T, GCIY, SNU-668, SNU-1 and FU97 were purchased from Cobioer (Nanjing). SNU-719, SNU-216, NCI-N87, AGS, HGC-27, MKN45, KATOIII, MKN1, 23132/87, SNU-16, HS746T, SNU-668 and SNU-1 were cultivated in RPMI-1640 medium (Gibco, CA, USA) supplemented with 10% fetal bovine serum (FBS) (Gibco, CA, USA). FU97 was cultivated in DMEM (Gibco, CA, USA) supplemented with 10% FBS. IM95 was cultivated in DMEM supplemented with 10% FBS and 10 mg/L insulin. GCIY was cultivated in DMEM supplemented with 15% FBS. All cancer cell lines were cultivated at 37 °C with 5% CO2. The human embryonic kidney cell line HEK293T was cultured in DMEM supplemented with 10% FBS at 37 °C with 10% CO2. Regular authentication of all cell lines was performed using STR (short tandem repeat) profiling at Genecarer company. All cell lines were tested quarterly using the Myco-Lumi Luminescent Mycoplasma Detection Kit (Beyotime, C0297M) and were consistently negative for mycoplasma.

### Establishment of PDOs

Fresh GC tissue samples were washed 3–5 times using cold DPBS (Thermo, 14190250) containing 2% penicillin/streptomycin (Thermo, 15140122) and 100 μg/ml Primocin (Invivogen, Ant-pm-2) and cut into small fragments (1–2 mm^3^) with sterile surgical scissors. The tissue was then digested with 10 μg/mL Liberase (Roche, 540115100) in 5 mL Advanced DMEM/F12 medium (Thermo, 12634010) containing 10 μM Rock inhibitor Y27632 (Sigma, Y0503) at 37 °C for 30 min. 500 μL FBS were added to terminate digestion. Resulting cell suspension was filtered through a 100 μm filter, and cells were collected through centrifugation at 1500 rpm for 5 min. Cells were then resuspended in Matrigel (RD, 3533-010-02) and seeded in a prewarmed 12-well plates. When the Matrigel was solidified, human gastric cancer organoid media was added [DMEM-F12 supplemented with 1% penicillin/streptomycin, 10 mM HEPES (Thermo, 15630080), 1% GlutaMAX (Thermo, 35050061), 1% N-2 supplement (Thermo, 17502-048), 1% B27 supplement (Thermo, 17504044), 100 μg/mL Primocin, 100 ng/ml Wnt3a (RD, 5036-WN), 50 ng/mL hEGF (PeproTech, AF-100-15), 100 ng/mL hFGF10 (PeproTech, 100-26), 0.5 μg/mL R-spondin (PeproTech, 120-38), 100 ng/mL Noggin (PeproTech, 250-38), 10 nM gastrin (Tocris Bioscience, 3006/1), 10 mM nicotinamide (Sigma Aldrich, 102413267), 1 mM N-acetyl-L-cysteine (Sigma Aldrich, A7250), 2 μM A83-01 (Tocris Bioscience, 2939/10), 3 μM SB202190 (Sigma Aldrich, S7067), 1 μM Prostaglandin E2 (Sigma Aldrich, P6532), and 3 μM Laduviglusib (MCE, HY-10182)]. 10 μM Rock inhibitor Y27632 was added for initial culture. Organoids were frozen in Cryopreservative Medium cellbanker 2 (ZENOAQ) and could be recovered efficiently.

### DNA constructs

The Constitutive Cas9 vector FUCas9Cherry (Addgene, 70182), the inducible guide RNA vector FgH1tUTG (Addgene, 70183), the constitutive guide RNA vector pKLV-U6gRNA (BbsI)-PGKpuro2ABFP (Addgene, 50946) as well as the 3rd generation lentiviral packaging plasmids pMDLg/pRRE (Addgene, 12251), pRSV-Rev (Addgene, 12253) and pCMV VSV-G (Addgene, 8454) were used in this study. The GV492 lentiviral expression constructs of human ELK1, ELK3, ELK4, STAT3, SRF, as well as the GV366 (C-terminally HA-tagged) and GV657 (C-terminally Flag-tagged) constructs for transient expression of human STAT3 and SRF were purchased from Shanghai Genechem Co., Ltd.

### Lentivirus production and infection

The lentivirus packaging plasmids pMDLg/pRRE, pRSV-Rev and pCMV VSV-G were transiently transfected into HEK293T cells with the construct of interest using Lipofectamine™ 3000 Transfection Reagent (Thermo, L3000015). Supernatant containing infectious virus particles was harvested 48 h later. A second viral harvest was made following a further 24 h incubation with fresh medium. Virus containing supernatant was filtered through a 0.45 µm filter and stored at 4 °C or −80 °C until used. Typically, GC cells were seeded into 6-well plates at 500,000 cells/well. An equivalent volume of viruses containing culture medium was added along with polybrene (Sigma) to a final concentration of 5 μg/mL. Cells were spin infected (1800 g, 25 °C, 1 h) and then incubated at 37 °C for 20 h. Cells were then washed and resuspended in fresh culture medium.

### Construction of MCL1 promoter constructs and Luciferase reporter assays

The human STAT3 and SRF ORFs were cloned into the GV657 expression vector (Shanghai Genechem Co., Ltd.). MCL1 promoter constructs were generated by inserting the promoter fragment of MCL1 (2000 bp upstream of the transcription start site, TSS), as well as the ones with putative binding sites of SRF or STAT3 mutated into the GV238 Luciferase Reporter vector (Shanghai Genechem Co., Ltd.). Luciferase activities were evaluated using the Dual-Luciferase Assay Kit (Promega, E2940) according to the manufacturer’s instructions.

### CRISPR/Cas9 gene editing

SNU-668, SNU-16, SNU-1, SNU-216, and 23132/87 were infected with lentiviruses expressing Cas9 (mCherry) and the pKLV-gRNAs targeting *BAX* and *BAK* (BFP). MCherry^+^BFP^+^ cells were sorted using BD FACSAriaTMII flow cytometer. Loss of BAX and BAK protein expression in the pooled cells were confirmed using Western blotting. To generated *BAX*^-/-^*BAK*^-/-^ or *FBXW7*^-/-^ single cell clones, MCherry^+^BFP^+^ cells were sorted into 96-well plates in one cell per well. Mutation of the targeted DNA was confirmed by targeted PCR followed by Sanger DNA sequencing. Single cell clones with frameshift mutations were used for this study. The inducible guide RNA vector FgH1tUTG was used to delete the pro-survival BCL2 family relatives, *ELK1*, *STAT3*, *SRF*, *NF-κB,* and *FBXW7*. To induce the expression of sgRNAs, doxycycline was added to cell culture medium at a final concentration of 1–2 μg/mL. The sequences of sgRNAs and primers are summarized in Table S2.

### Compounds and reagents

Venetoclax, A1331852, S63845, S64315, irinotecan, cisplatin, carboplatin, paclitaxel, docetaxel, vincristine, 5-FU, doxycycline, MG132, chloroquine, Q-VD-Oph, trastuzumab, lapatinib, as well as the STAT3 inhibitor BBI608 were purchased from MedChemExpress (MCE). Thymidine and Propidium Iodide (PI) were purchased from Sigma-Aldrich.

### Cell viability assays

To test the response of GC cells to BH3-mimetics, cells were seeded in 96-well plates at 3 × 10^3^ cells/well and treated with titrated concentrations (0–10 µM) of indicated drugs for 24 h. Similar experiments were performed with anti-mitotic chemotherapies and HER2-targeting drugs with cell viability tested at 24–96 h after treatment. Cell viability was determined using the CellTiter-Glo 2.0 Cell Viability Assay (Promega, G9242) according to the manufacturer’s instructions. The percentage of cell viability was calculated by normalizing to that observed in cells treated with the vehicle control DMSO. GraphPad Prism software was used to calculate drug concentrations causing 50% killing (IC50).

To measure the percentage of cell death induced by BCLXLi, anti-mitotic drugs or their combinations, 1 × 10^4^ WT, sg*EV,* and *BAX*/*BAK* deficient subclone of SNU-216 cells were seeded in 12-well plates and treated with drugs for 72 h. Cell viability was determined by PI staining (2.5 μg/mL), followed by flow cytometry analysis. The FlowJo software was used to analyze the data. The percentage of PI positivity was calculated by normalizing to the cells treated with the DMSO control.

To test the synergistic activity of drug combinations comprehensively, cells were seeded in 96-well plates at 3 × 10^3^ cells/well and treated with BH3 mimetics individually and in a combination matrix that paired every concentration of both drugs. The predicted additive effect of combining two drugs was calculated using the BLISS model of fractional independence [63] and subtracted from the actual measured combinatorial effect to generate BLISS scores for each combination at different drug concentrations. BLISS values > 0.0 indicate synergy between the two drugs at indicated concentrations.

To test the viability of PDOs treated by BH3-mimetics or in combination with docetaxel, PDOs were dissociated into single cells and 5 × 10^3^ cells were seeded per dome in 96-well plates. The cells were then allowed to grow into organoids over a period of 7–9 days. Subsequently, the culture medium of the organoids was replaced with medium containing different concentrations of drugs along with 10 μg/mL PI.

Cell viability was determined using the CellTiter-Glo 3D Cell Viability Assay (Promega, G9682) according to the manufacturer’s instructions 72 h after treatment. Bright field and fluorescent images were taken using the Leica DMi 8 microscope 72 h after treatment.

### Cell cycle synchronization

Cells were seeded in 10 cm^2^ plates at 1 × 10^6^ cells/plates. Cell synchronization was achieved by culture in medium containing 2 mM thymidine for 20 h, release from the thymidine block with three washes in PBS, followed by culture in complete growth media for another 12 h. Cells then underwent a second thymidine block for 20 h, followed by three further washes in PBS. Synchronized cells were then released into complete medium containing the indicated reagents.

### In vivo studies

To generate NCI-N87 and SNU-668 xenografts models, GC cells were harvested during exponential growth and 5 × 10^6^ cells suspended in 100 μl PBS were inoculated into the right flank of BALB/c nude mouse subcutaneously. Mice were randomized when tumors reached a volume of ~100 mm^3^. After randomization, mice were treated with BH3-mimetic drugs alone or in combination with docetaxel or HER2-targeting drugs. BCLXL inhibitor was formulated for oral dosing in 60% phosal 50 propylene glycol (PG), 27.5% polyethylene glycol (PEG) 400, 10% ethanol, and 2.5% DMSO, MCL1 inhibitor was formulated for i.v. injection in 2% Vitamin E. Trastuzumab was formulated for i.p. injection in PBS. Docetaxel and lapatinib were formulated for i.p. injection in 45% PBS, 27.5% PEG 400, 25% Tween-80 and 2.5% DMSO. Tumor volumes and mouse weights were measured every 3 days. Tumor volumes were calculated using the formula: volume = 1/2 × length × width × width.

To test the combined effect of targeting BCLXL and MCL1 in vivo, 5 × 10^6^ HGC-27 cells expressing the sgRNA empty vector or the subclones expressing the inducible sgRNA targeting BCLXL were suspended in 100 μL PBS and inoculated into the right flank of BALB/c nude mouse subcutaneously. When tumors reached a volume of ~100 mm^3^, mice were replaced with doxycycline-containing food alone or together with MCL1 inhibitor treatment.

For the establishment of PDX models, fresh tumor tissue fragments (1–2 mm^3^) were implanted into both flanks of female NTG mice subcutaneously. When tumors grew to a size of about 1 cm^3^, they were carefully excised and minced into 1–2 mm^3^ pieces for passaging. Following the same procedure, minced tumor fragments were re-implanted into the right flank of NTG mice. Mice were monitored and randomized once the tumor volume reached to ~100 mm^3^. After randomization, mice were treated with BH3-mimetic drugs alone or in combination with docetaxel. No blinding was done during the animal experiments.

### RNA isolation and qRT-PCR analysis

Total RNA was extracted from cells using TRIzol (Thermo Fisher Scientific, 15596026). cDNA was then synthesized using the PrimeScript™ RT reagent Kit (TaKaRa, RR037B) according to the manufacturer’s instructions. qRT-PCR was conducted using the TB Green Premix Ex Taq II (Tli RNaseH Plus) (TaKaRa, RR82WR) according to the manufacturer’s instructions. Data were presented as the relative level of MCL1 mRNA normalized to GAPDH. The sequences of qRT-PCR primers are summarized in Table S2.

### Western blotting

Total protein was extracted using RIPA buffer containing complete protease inhibitors (Roche, 4693132001). The protein concentration in the lysates was quantified using the Enhanced BCA Protein Assay Kit (Beyotime, P0010S). Lysates were diluted with 5 × SDS-PAGE protein loading buffer at a 4:1 ratio and denatured by boiling at 95 °C for 10 min. 40–60 µg denatured proteins were loaded per well and separated by electrophoresis through an 10% SDS-PAGE gel. Quantification of western blots was performed using the ImageJ software and data were normalized to the expression of loading control β-actin or GAPDH. Blots in the study are representative of 2 independent experiments. The primary and HRP-conjugated secondary antibodies used in this study were summarized in Table S3. Full length Western blots are provided in supplementary information.

### Co-immunoprecipitation (Co-IP)

4x10^7^ cells per sample were lysed in 500 μL lysis buffer. Pre-cleared lysates were then incubated with indicated antibody at 4 °C for 3 h followed by incubation with protein A/G agarose beads (Beyotime, P2029) at 4 °C overnight. Beads were then collected by centrifugation at 3000 rpm at 4 °C for 1 min and washed with PBS for 10 times. Bound proteins were eluted with 50 μL 2 × SDS reducing buffer and denatured by boiling at 95 °C for 10 min. Immunoprecipitation (IP) and input (Input) samples were analyzed by immunoblotting.

### Chromatin immunoprecipitation (ChIP)

ChIP was performed using the ChIP assay kit according to the manufacturer’s instructions (Beyotime Biotechnology, P2080S). Briefly, SNU-216 cells were treated with 100 ng/ml trastuzumab or 200 nM lapatinib for 72 h and 48 h respectively. Subsequently, they were cross-linked by incubated with 1% formaldehyde (final concentration) for 10 min at room temperature, and neutralized by the addition of 125 mM glycine (final concentration) for 5 min. Cells were lysed and sonicated to produce DNA fragments 400–800 bp in length using the Bioruptor sonication system (Epishear, 53052) following the manufacturer’s instructions. The supernatants were used as the chromatin input. Antibodies for STAT3(CST, 79D7, Rabbit), SRF (CST, D71A9, Rabbit) were used to perform immunoprecipitation. Protein A/G Magnetic Beads/Salmon Sperm DNA was added and incubated at 4 °C for 60 min. BeyoMag™ Rabbit IgG Magnetic Beads (Beyotime Biotechnology, P2173) were used as the negative control. The bound complex containing DNA, protein, and antibody was precipitated using magnetic rack, washed with washing buffer, eluted with elution buffer containing 0.1 M NaHCO3 and 1% SDS, and de-crosslinked at 65 °C for 4 h in the presence of 0.2 M NaCl (final concentration). The retrieved DNA was purified using the ChIP DNA Clean & Concentrator (ZYMO RESEARCH, D5201). The purified DNA was subsequently used for PCR detection. The sequences of PCR primers are summarized in the Table S2.

### Statistical analysis

GraphPad Prism software v10.1 was used for statistical analysis. Unpaired Student’s t test was used for comparison between the two groups. One-way ANOVA analysis was used to compare more than two groups, and correction for multiple comparisons was applied when needed. Spearman’s rank correlation coefficient analysis was used to calculate the correlation between the expression levels of BCL2 family proteins/STAT3 and IC50s to BH3-mimetc drugs or the STAT3 inhibitor. The genomic mutation, copy number variation, and MSI status data of the GC cell lines were sourced from the TCGA PanCancer Atlas study [64] using the cBioPortal [65]. The Wilcoxon rank sum test [66] was used to assess the association between the response of gastric cancer cell lines to BH3-mimetic drugs and genetic alterations implicated in GC tumorigenesis and the Complex Heatmap package was used for data visualization [67]. Data are reported as mean ± SD of ≥3 independent experiments and threshold for significance was *p* < 0.05. *p < 0.05; **p < 0.01; ***p < 0.001.

## Supplementary information

Zhang et al Supplementary Information-Clean

Table S1. Patient information

Table S2. Oligonucleotides used in this study

Table S3. Antibodies used in this study-Revised

Original Western Blot and ChIP PCR data

Reproducibility checklist

## Data Availability

All the raw data generated in this study are available upon request from the corresponding author.
