# Supplementary material for: Both direct and indirect suppression of MCL1 synergizes with BCLXL inhibition in preclinical models of gastric cancer
Source: Cell Death Dis. 2025 Mar 12;16(1):170. doi: 10.1038/s41419-025-07481-8 (PMC11904182; doi:10.1038/s41419-025-07481-8)
Supplement: Supplementary file 1 — Zhang et al Supplementary Information-Clean [file 41419_2025_7481_MOESM1_ESM.docx]

**Supplementary information**

**Table S1.** Patient information

**Table S2.** Oligonucleotides used in this study

**Table S3.** Antibodies used in this study

**Supplementary figures and legends**

**Supplementary figures and legends**


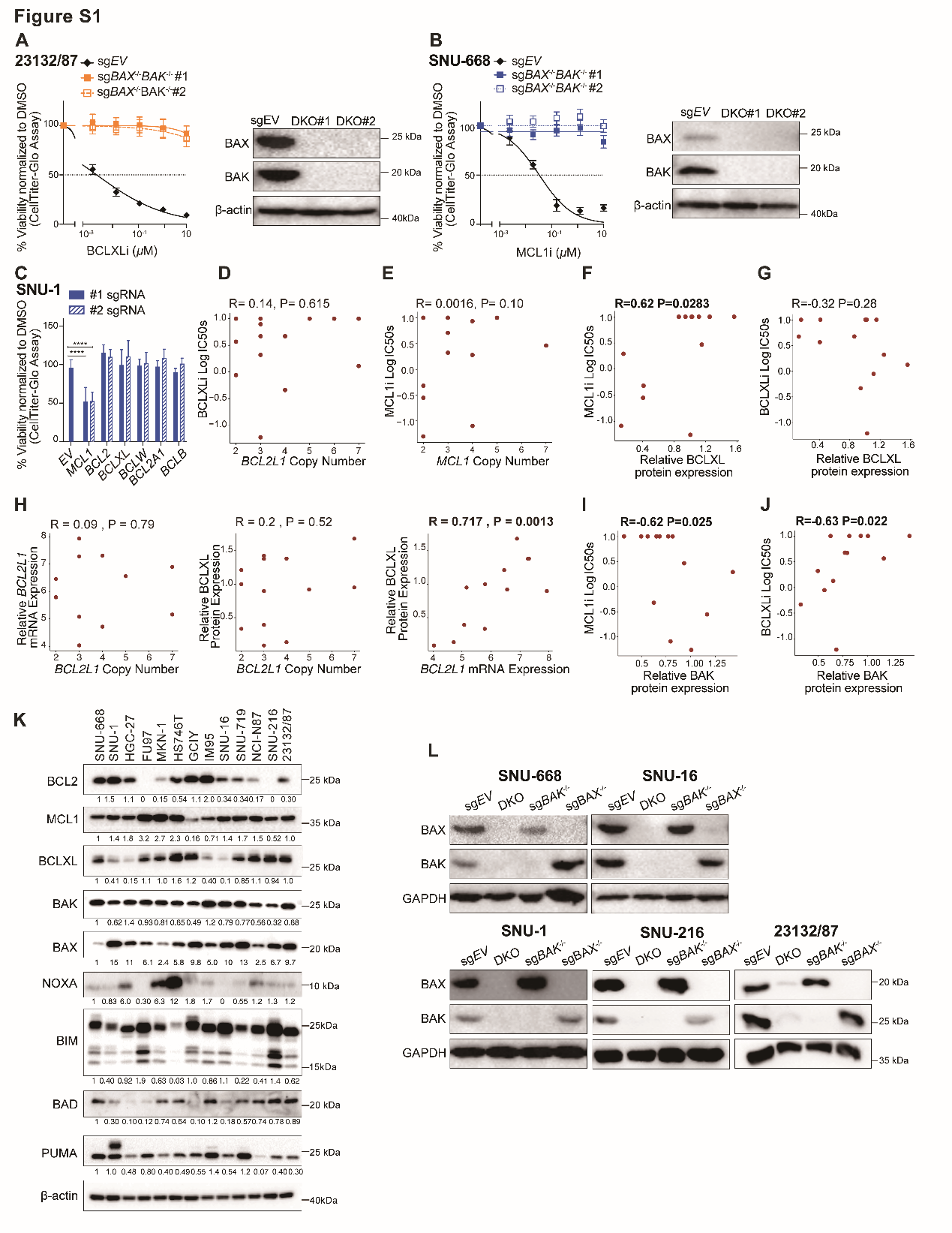


**Figure S1. Related to Figure 1.**

(**A, B**) BAX/BAK-dependent killing by BCLXLi and MCL1i. The viability of WT and *BAX*^-/-^*BAK*^-/-^ subclones of 23132/87 (A) or SNU-668 (B) cells 24 h after treatment with 0-10 μM BCLXLi or MCL1i was determined.

(**C**) Genetic deleting *MCL1* induces cell death in SNU-1 cells. Cell viability 72 h after addition of DOX to induce the expression of sgRNAs that target *BCL2*, *BCLXL*, *BCLW*, *MCL1*, *BCL2A1* or *BCLB* was determined. 2 sgRNAs were tested for each gene. ­

(**D**) Spearman’s correlation analysis between *BCL2L1* CNVs and BCLXLi sensitivity.

(**E**) Spearman’s correlation analysis between *MCL1* CNVs and MCL1i sensitivity.

(**F**) Spearman’s correlation analysis between BCLXL protein levels and MCL1i sensitivity.

(**G**) Spearman’s correlation analysis between BCLXL protein levels and BCLXLi sensitivity.

(**H**) Spearman’s correlation analysis between *BCL2L1* CNVs, mRNA and BCLXL protein levels.

(**I**) Spearman’s correlation analysis between BAK protein levels and MCL1i sensitivity.

(**J**) Spearman’s correlation analysis between BAK protein levels and BCLXLi sensitivity.

(**K**) Quantification of the expression levels of BCL2 family proteins shown in Figure 1I. The signal intensity of protein bands was analyzed using the ImageJ software and bands for the indicated proteins were normalized to the corresponding loading control β-actin. The expression levels relative to the SNU-668 sample were displayed.

(**L**) Expression of BAX and BAK in pools of indicated GC lines expressing sgRNAs targeting *BAX*, *BAK* or both.

Cell viability was determined using the CellTiter‑Glo assay; cell viability data in panel (**A-C**) represent the means ± SD of ≥ 3 independent experiments; blots in panel (**A**), (**B**), (**K**) and (**L**) are representatives of 2 independent experiments.


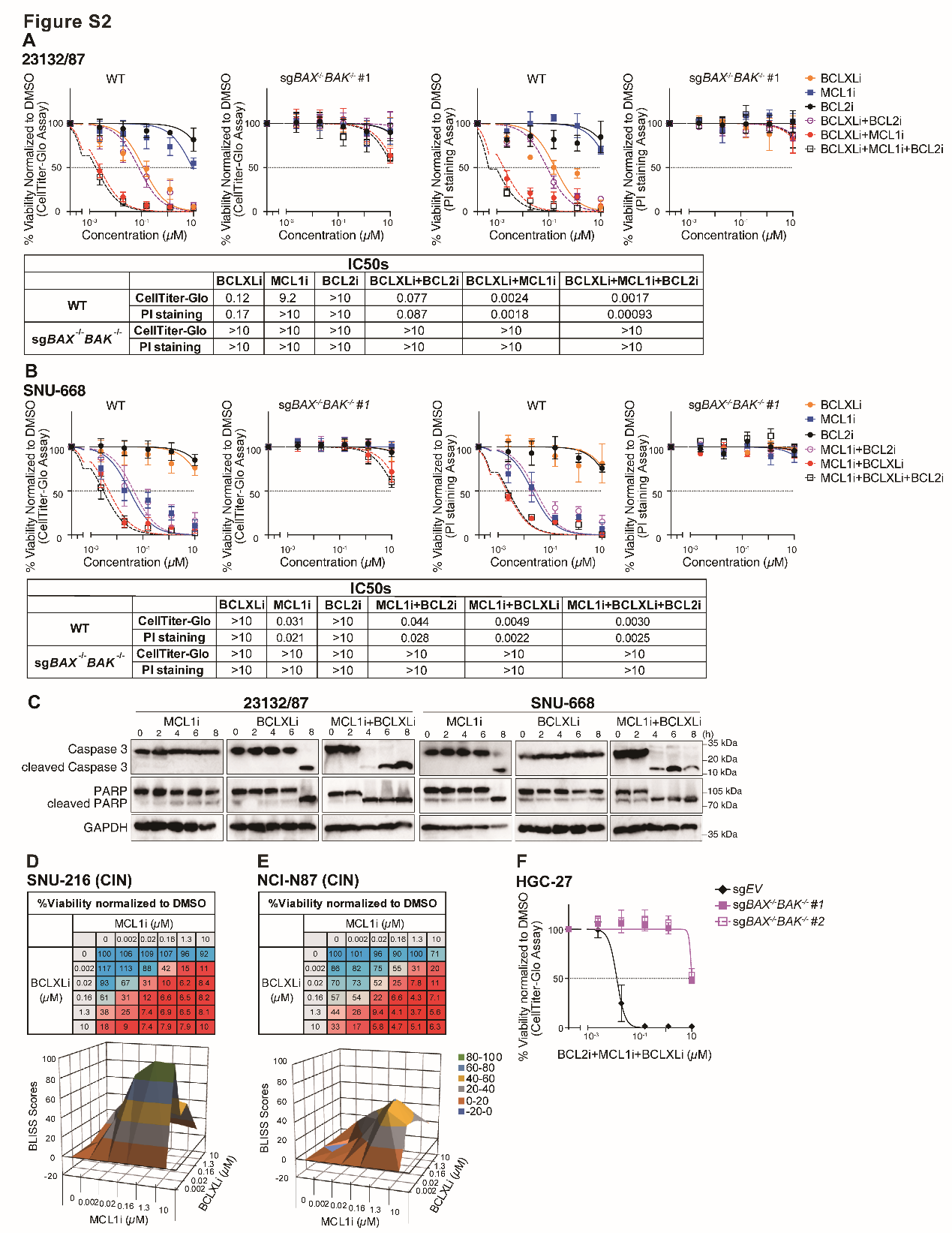


**Figure S2. Related to Figure 2.**

(**A, B**) Comparison between CellTiter-Glo and PI staining assays. WT and *BAX/BAK* deficient subclone of 23132/87 (A) and SNU-668 (B) cells were treated with titrated concentrations (0-10 μM) of BH3-mimetic drugs either alone or in combinations for 48 h and the viability was measured in parallel by CellTiter-Glo or PI staining followed by Flow cytometric analysis. The mean values of IC50s ± SD of 3 independent experiments were summarized in the table.

(**C**) Accelerated caspase 3 and PARP cleavage with dual inhibition of BCLXL and MCL1. WT 23132/87 and SNU-668 cells were treated with MCL1i (100 nM), BCLXLi (100 nM) or both (100 nM for both drugs) for the indicated time periods. The protein levels of full-length and cleaved caspase 3 and PARP were determined.

(**D, E**) Synergistic killing by dual inhibition of BCLXL and MCL1 in SNU-216 (D) and NCI-N87 (E) cells.

(**F**) BAX/BAK dependent killing of the triple combinations of MCL1i, BCLXLi, and BCL2i. The viability of WT and *BAX*^-/-^*BAK*^-/-^ subclones of HGC-27 cells 24 h after treatment with the triple combination of MCL1i, BCLXLi and BCL2i (0-10 μM, 1:1:1) was determined.

Blots in panel (**C**) are representatives of 2 independent experiments; cell viability in panel (**D-F**) was determined using the CellTiter‑Glo assay; cell viability data in panel (**A-B**) and (**D-F**) represent the means ± SD of ≥ 3 independent experiments.


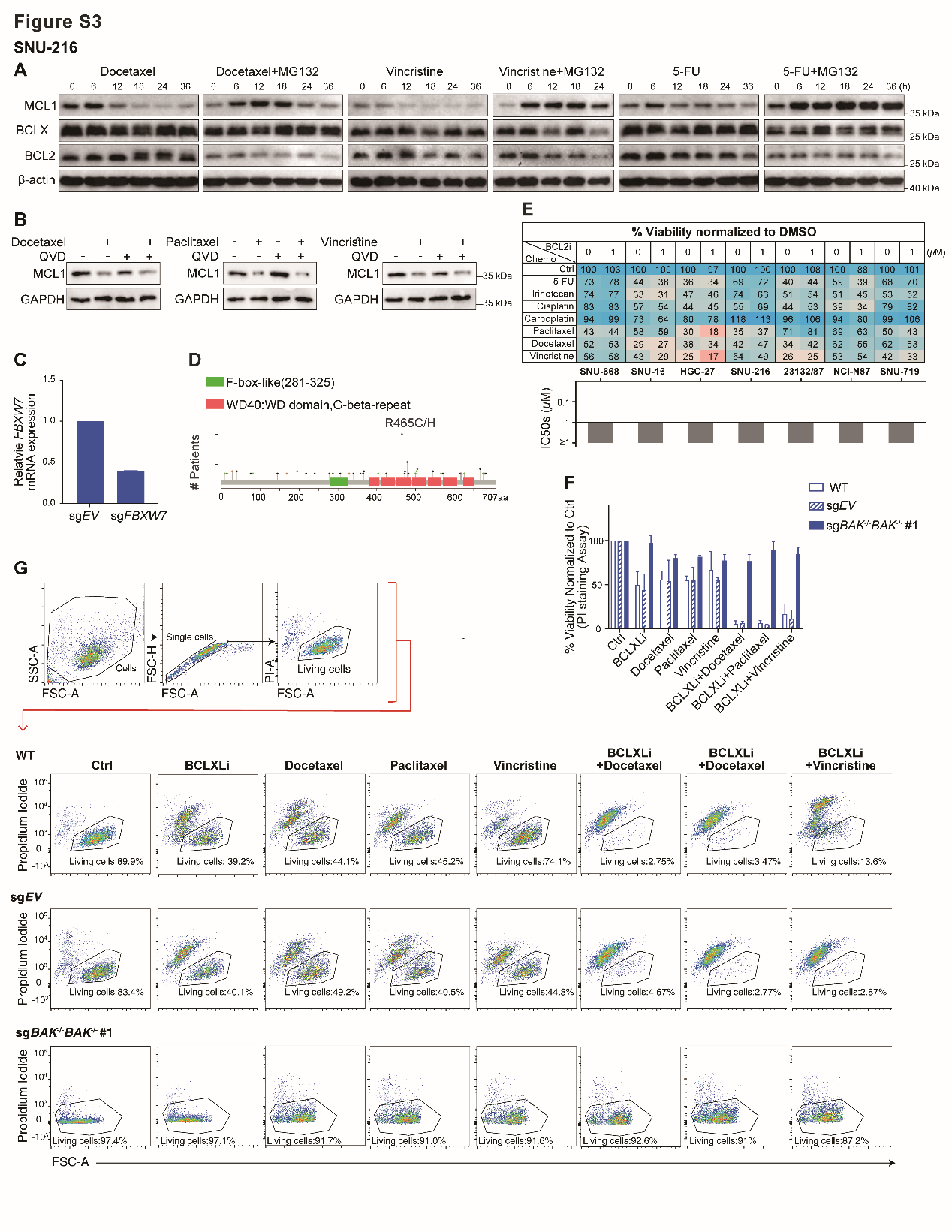


**Figure S3. Related to Figure 3.**

(**A**) Anti-mitotic drugs, but not 5-Fu, induced rapid reduction of MCL1 proteins levels in SNU-216 cells. Synchronized SNU-216 cells were treated with 10 μM docetaxel, vincristine or 5-Fu alone or in combination with 5 μM MG132 for indicated time periods and the protein levels of MCL1, BCLXL and BCL2 were determined.

(**B**) Western blotting analysis of MCL1 protein levels in SNU-216 cells 24 h after treatment with anti-mitotic drugs alone (10 μM) or together with the pan-caspase inhibitor Q-VD-Oph (QVD, 10 μM).

(**C**) Validation of target deletion in SNU-216 cells expressing sg*FBXW7* by qRT-PCR.

(**D**) Analysis of *FBXW7* mutation in primary GC patient samples using the cbioportal database.

(**E**) *In vitro* activity of BCL2i in combination with different chemotherapies. The responses of indicated GC cell lines to BCL2i alone or in combination with different chemotherapies 72 h after treatment were determined using the CellTiter‑Glo assay.

(**F**) WT, sg*EV* and *BAX*/*BAK* deficient subclone of SNU-216 cells were treated with BCLXLi (1 μM), either of the anti-mitotic drugs (10 μM) or in combinations for 72 h and cell viability was determined by PI staining followed by Flow cytometric analysis. The percentage of PI negativity was calculated by normalizing to the cells treated with the DMSO control.

(**G**) Gating strategy and representative flow cytometric plots from panel (F).

Blots in panel (**A**) and (**B**) are representatives of 2 independent experiments; data in panel (**C**), (**E**) and (**F**) represent the means ± SD of ≥ 3 independent experiments.

**
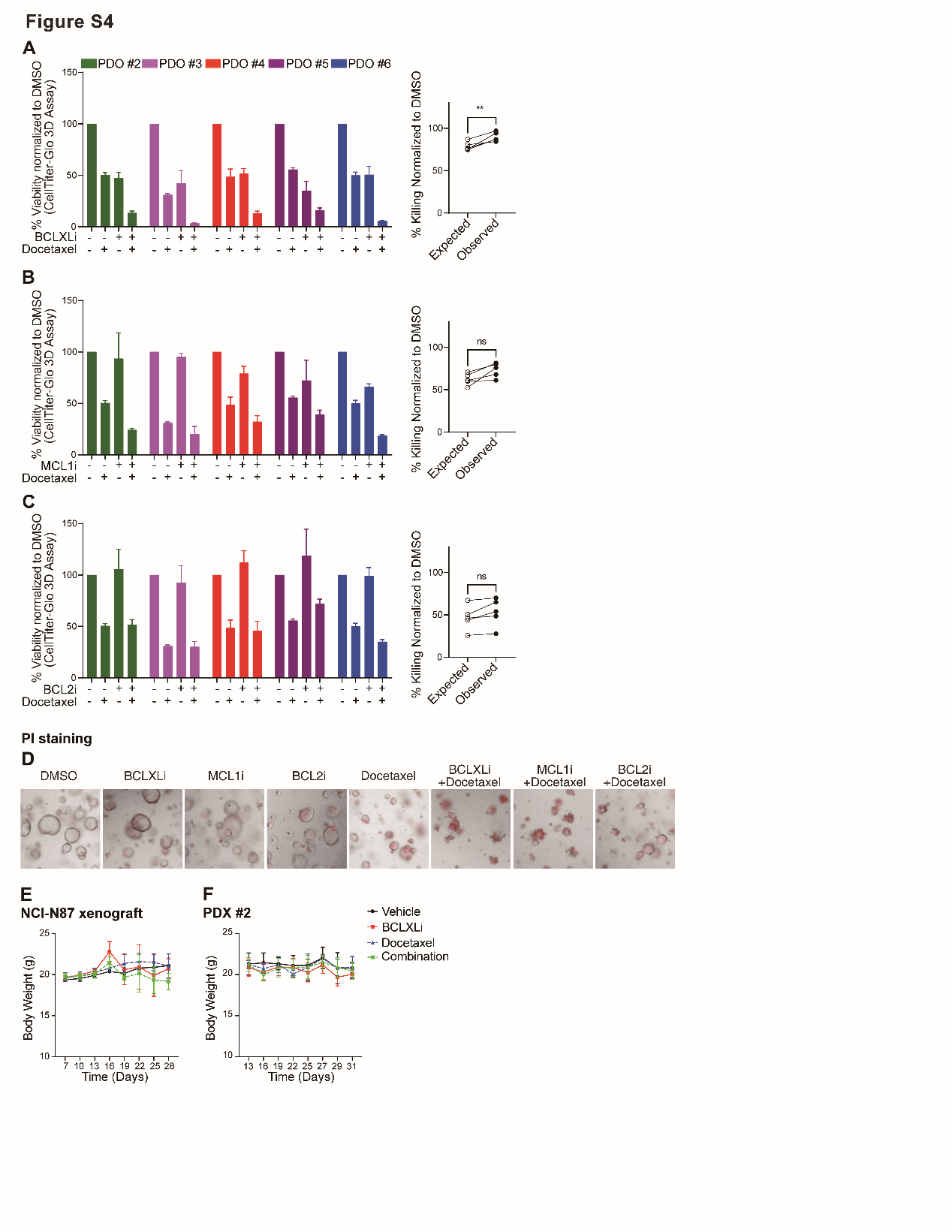
**

**Figure S4. Related to Figure 4.**

(**A-C**) Similar experiments to those in Figure 4A-4C were performed with higher doses of BH3-mimetic drugs. The concentrations of BCLXLi, MCL1i and BCL2i used: PDO #2, #3, #5: 100 nM, PDO #4, #6: 1 *μ*M.

(**D**) The responses of PDO #6 to indicated treatments were determined by PI (red) staining. Representative images at 72 h after treatment were shown.

(**E, F**) Effect of combination therapy on mouse weights.

Cell viability was determined using the CellTiter‑Glo 3D assay; data in panel (**A-C**) represent the means ± SD of ≥ 3 independent experiments.


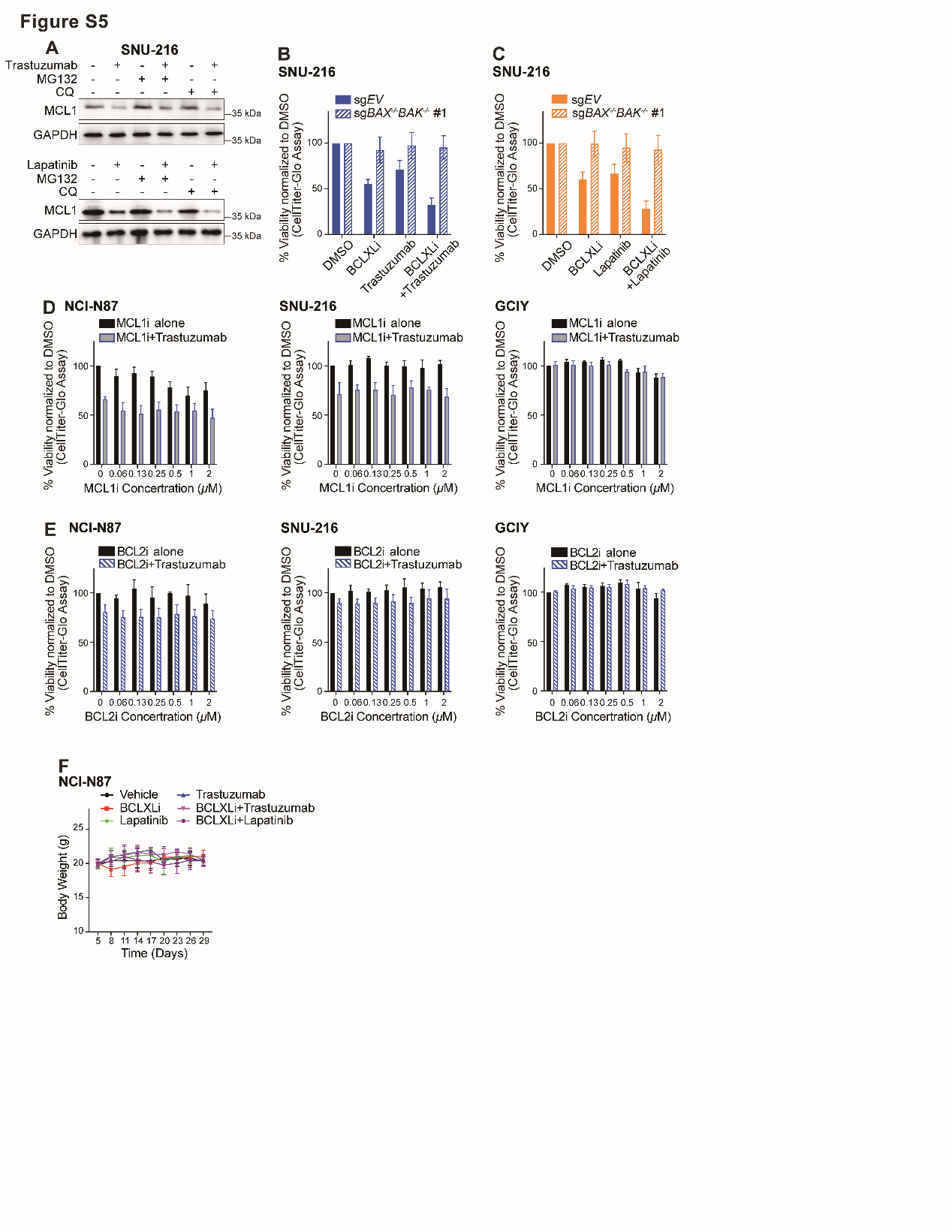


**Figure S5. Related to Figure 5.**

(**A**) Blocking ubiquitin- and lysosome-mediated protein degradation failed to revert the decrease of MCL1 expression. SNU-216 cells were treated with 100 ng/ml trastuzumab, 200 nM lapatinib alone or together with 5 μM MG132 or CQ for 72 h or 48 h respectively. The protein levels of MCL1 were determined by Western blotting. Blots are representatives of 2 independent experiments.

(**B, C**) Loss of *BAX* and *BAK* efficiently blocked cell death induced by BCLXLi and HER2-targeting drugs. The viability of WT and *BAX*^-/-^*BAK*^-/-^ subclones of SNU-216 cells 72 h after treatment with 1 μM BCLXLi, 100 ng/ml trastuzumab (B) or 200 nM lapatinib (C) alone or in combinations was determined.

(**D, E**) Inhibiting MCL1 or BCL2 has no effect on enhancing the activity of trastuzumab. The cell viability of NCI-N87, SNU-216 or GCIY 72 h after treatment with 100 ng/ml trastuzumab ­alone or in combination with indicated concentrations of BCL2i or MCL1i was determined.

(**F**) Effect of combination therapy on mouse weights.

Cell viability was determined using the CellTiter‑Glo assay; data in panel (**B-E**) represent the means ± SD of ≥3 independent experiments.


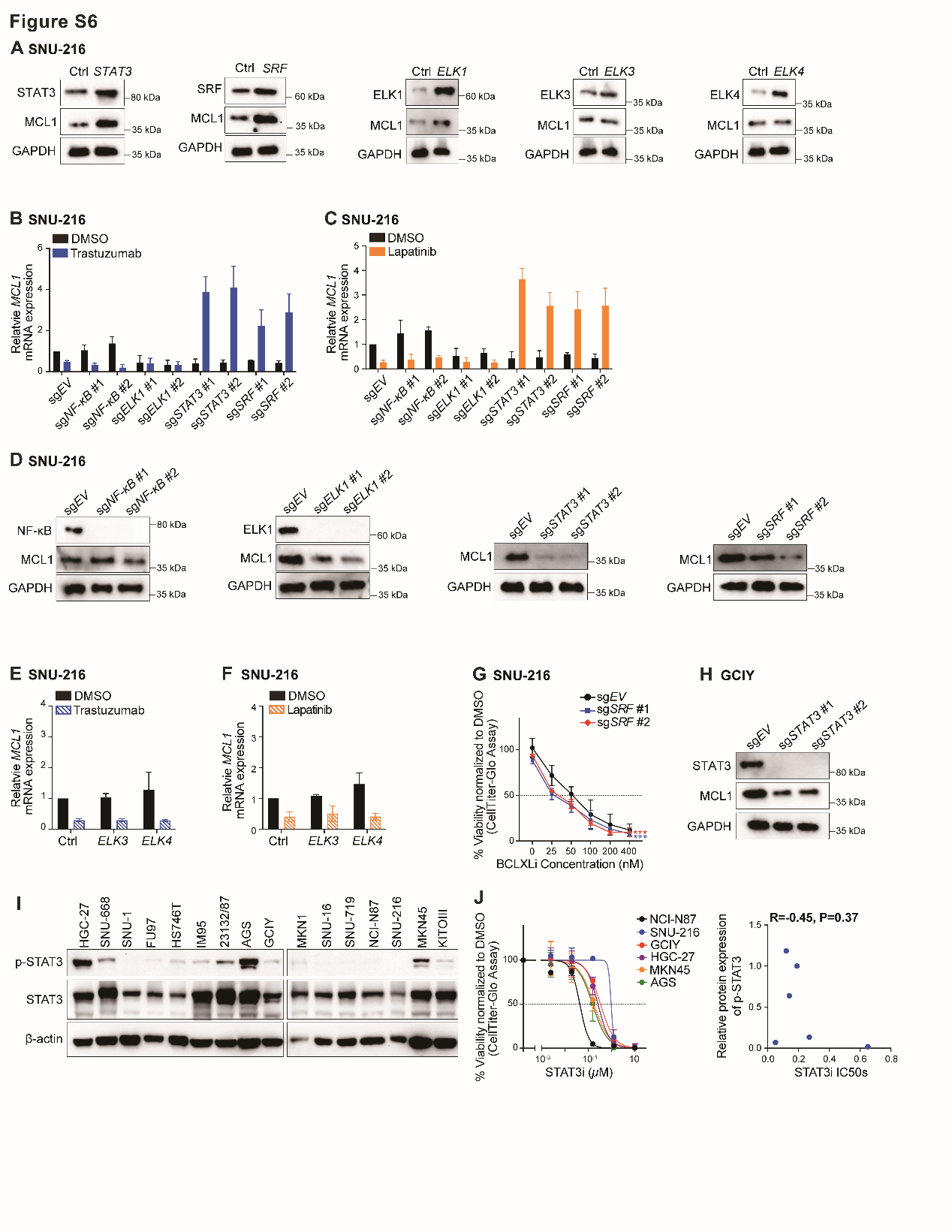


**Figure S6. Related to Figure 6.**

(**A**) Validation of target overexpression and MCL1 protein levels in SNU-216 cells expressing the empty vector, *STAT3*, *SRF*, *ELK1*, *ELK3* and *ELK4*.

(**B, C**) Role of NF-κB, ELK1, STAT3 and SRF in regulating the transcriptional activity of MCL1 under basal condition and in response to HER2-targeting drugs. SNU-216 cells expressing the sgRNA empty vector or sgRNAs targeting the indicated transcriptional factors were treated with 100 ng/ml trastuzumab or 200 nM lapatinib for 72 h or 48 h, respectively. The mRNA levels of *MCL1* were determined using RT-qPCR. 2 independent sgRNAs for each gene were used.

(**D**) Validation of target deletion and MCL1 protein levels in SNU-216 cells expressing sgRNAs targeting *NF-κB*, *ELK1*, *STAT3* or *SRF*.

(**E, F**) Role of ELK3 and ELK4 in regulating the transcriptional activity of *MCL1* under basal condition and in response to HER2-targeting drug treatment. Similar experiments to those in panel (A) and (B) were performed with vectors expressing *ELK3* or *ELK4*.

(**G**) Depletion of *SRF* increased the sensitivity of GC cell lines to BCLXL inhibition. SNU-216 cells inducibly expressing sg*SRF* or the sgRNA empty vector were treated with DOX alone, or in combination with indicated concentrations of BCLXLi for 48 h. Cell viability was determined using the CellTiter‑Glo assay.

(**H**) Validation of target deletion and MCL1 protein levels in GCIY cells expressing sgRNAs targeting *STAT3*.

(**I**) Protein levels of p-STAT3 and STAT3 in the panel of GC lines.

(**J**) The responses of GC cell lines to the STAT3 inhibitor BBI608 were independent of their p-STAT3 level. GC cell lines with highest p-STAT3 expression (HGC-27, MKN45 and AGS) and the ones with least p-STAT3 expression (SNU-216, NCI-N87, GCIY) were treated with the indicated concentrations of STAT3 inhibitor BBI608 for 48 h. Cell viability was determined using the CellTiter‑Glo assay. Left panel: Spearman’s correlation analysis between p-STAT3 protein levels and STAT3i sensitivity.

Blots in panel (**A**), (**D**), (**H**) and (**I**) are representatives of 2 independent experiments; data in panel (**B**), (**C**), (**E-G**) and (**J**) represent the means ± SD of ≥ 3 independent experiments.
