# Supplementary material for: Both direct and indirect suppression of MCL1 synergizes with BCLXL inhibition in preclinical models of gastric cancer
Source: Cell Death Dis. 2025 Mar 12;16(1):170. doi: 10.1038/s41419-025-07481-8 (PMC11904182; doi:10.1038/s41419-025-07481-8)
Supplement: Supplementary file 2 — Table S1. Patient information [file 41419_2025_7481_MOESM2_ESM.pdf]

Table S1. Patient information

Patient ID	Study ID	Gender	Diagnosis Age(y)	Pathological diagnosis	Lauren subtype	TNM stages	Previous Rx	Tumor site	TP53 mutation	MSI/MSS status
P1	PDO#1	Female	42	Poorly differentiated adenocarcinoma	Mixed	cT4aN3bM0 IIIB	FOLFOX4	Corpus	90%+	MSI
P2	PDO#2	Female	47	Poorly differentiated adenocarcinoma	Diffuse	cT4aN1M0 IIIA	nil	antrum	40%+	MSS
P3	PDO#3	Male	58	Poorly differentiated tubular adenocarcinoma	Mixed	cT4aN3aM0 IIIB	nil	antrum	90%+	MSS
P4	PDO#4	Male	42	Poorly differentiated adenocarcinoma	Diffuse	cT4aN2M0 IIIB	nil	antrum	WT	MSS
P5	PDO#5	Male	53	Poorly differentiated adenocarcinoma	Diffuse	cT3N3aM0 IIIB	nil	Corpus	80%+	MSS
P6	PDO#6	Female	49	Poorly differentiated tubular adenocarcinoma	Intestinal	cT4aN1M0 IIIB	nil	antrum	80%+	MSI
P7	PDX#1	Male	66	Poorly differentiated mucinous adenocarcinoma	Diffuse	cT4aN2M1 IVB	nil	antrum	<20%+	MSS
P8	PDX#2	Male	59	Poorly differentiated adenocarcinoma	Intestinal	cT3N3M0 IIIB	Sox	antrum	ND	ND

ND: not determined
