# Supplementary material for: Both direct and indirect suppression of MCL1 synergizes with BCLXL inhibition in preclinical models of gastric cancer
Source: Cell Death Dis. 2025 Mar 12;16(1):170. doi: 10.1038/s41419-025-07481-8 (PMC11904182; doi:10.1038/s41419-025-07481-8)
Supplement: Supplementary file 3 — Table S2. Oligonucleotides used in this study [file 41419_2025_7481_MOESM3_ESM.pdf]

Table S2. Oligonucleotides used in this study

**Sequences of sgRNAs**
h*MCL1* sgRNA#1: GGGAGGGCGACTTTTGGCTA
h*MCL1* sgRNA#2: GGAGCTGGACGGGTACGAGC
h*BCL2* sgRNA#1: GCGGCGGGAGAAGTCGTCGC
h*BCL2* sgRNA#2: GAGAACAGGGTACGATAACC
h*BCL2L1* sgRNA#1: GGCCTTTTTCTCCTTCGGCG
h*BCL2L1* sgRNA#2: GTTTGAACTGCGGTACCGGC
h*BCLW* sgRNA#1: GAAAAAGTTCATCGGAGACC
h*BCLW* sgRNA#2: GGAGTTCACAGCTCTATACG
h*BCL2A1* sgRNA#1: CTTATAGGTATCCACATCCG
h*BCL2A1* sgRNA#2: GTCCTACAGATACCACAACC
h*BCLB* sgRNA#1:GCAACTGGTCAACCATGGTC
h*BCLB* sgRNA#2: GTAGTCGGCCAGCAACAGCT
h*BAX* sgRNA #1: CTGCAGGATGATTGCCGCCG
h*BAX* sgRNA #2: TCTGACGGCAACTTCAACTG
h*BAK* sgRNA #1: GCATGAAGTCGACCACGAAG
h*BAK* sgRNA #2: GGCCATGCTGGTAGACGTGT
h*STAT3* sgRNA#1: AGATTGCCCGGATTGTGGCC
h*STAT3* sgRNA#2: GCAGCTTGACACACGGTACC
h*ELK1* sgRNA#1: GTGTAGCGTGGCGGTGGCGT
h*ELK1* sgRNA#2: GGAATAAATAAGGCCACGGA
h*NF-κB* sgRNA#1: CGTCCACGTAAGTCAACCAC
hNF-κB sgRNA#2: TGATGTCATAAGTAACCCGC
h*SRF* sgRNA#1: CGGAATCGCGGTCTGACGCG
h*SRF* sgRNA#2: TTTAGTAACAGCGCGAGTGC
h*FBXW7* sgRNA:GATCAAAATCGTCACTCTCC
**Sequencing primers**
h*BAX* sgRNA#1 sequencing primer-F:CTTTAGTGTGCGGTGGATGC
h*BAX* sgRNA#1 sequencing primer-R:CCTTGAGCACCAGTTTGCTG
h*BAK* sgRNA#1 sequencing primer-F: CTATGGGATGCTCTGCCCAC
h*BAK* sgRNA#1 sequencing primer-R: GGTCACAGAGAGGCTAGCAG
h*FBXW7* R465C sequencing primer-F: AGTGGGACATACAGGTGGAG
h*FBXW7* R465C sequencing primer-R: AGAAAGGGCCCAAATTCACC
**Primers for qRT-PCR**
h*GAPDH*- F:GCACCGTCAAGGCTGAGAAC
h*GAPDH* -R:TGGTGAAGACGCCAGTGGA

h*MCL1* -F:AGAAAGCTGCATCGAACCAT
h*MCL1* -R:CCAGCTCCTACTCCAGCAAC
h*FBXW7* -F:CCATGCAAAGTCTCAGAATATACAAG
h*FBXW7* -R:TGGACAGATGTAATTCGGCG
**Primers for ChIP assays**
h*MCL1* -F:ACAGAGGTAGCCACGAGAAGG
h*MCL1* -R:TGGAAGGAAGCGGAAGTGAGAAG
h*SRF* -F: TCCGGTTCCTTTAAGAGGCG
h*SRF* -R:TACCGAACTCGTTGCTGTCA
