# Supplementary material for: Both direct and indirect suppression of MCL1 synergizes with BCLXL inhibition in preclinical models of gastric cancer
Source: Cell Death Dis. 2025 Mar 12;16(1):170. doi: 10.1038/s41419-025-07481-8 (PMC11904182; doi:10.1038/s41419-025-07481-8)
Supplement: Supplementary file 4 — Table S3. Antibodies used in this study-Revised [file 41419_2025_7481_MOESM4_ESM.pdf]

Table S3. Antibodies used in this study

Primary antibodies				
Antigen	Host species	Dilution	Source	Catolog number
BCL2	Rabbit	1:1000	abcam	ab182858
BCLXL	Rabbit	1:1000	abcam	ab32370
MCL1	Rat	1:1000	abcam	ab243136
BAX	Rabbit	1:1000	CST	5023S
BAK	Rabbit	1:1000	CST	12105S
BIM	Rabbit	1:1000	CST	2933S
BAD	Rabbit	1:1000	CST	9239S
PUMA	Rabbit	1:1000	CST	98672S
NOXA	Rabbit	1:1000	CST	14766S
Caspase-3	Rabbit	1:1000	CST	9662
PARP	Rabbit	1:1000	CST	9542
STAT3	Rabbit	1:1000	CST	79D7
STAT3	Mouse	1:1000	CST	9139
p-STAT3	Rabbit	1:1000	CST	9145
SRF	Rabbit	1:1000	CST	D71A9
ELK1	Rabbit	1:1000	CST	9182
NF-κB	Rabbit	1:1000	CST	8242
ELK3	Mouse	1:1000	Novus	NBP2-01264
ELK4	Rabbit	1:1000	Novus	NBP1-87092
HSP70	Mouse	1:1000	OriGene	TA-11
β-actin	Mouse	1:1000	OriGene	TA-09
GAPDH	Mouse	1:1000	OriGene	TA-08
Anti-HA tag	Mouse	1:1000	Sino Bilogical	100028-MM10
Anti-Flag tag	Rabbit	1:1000	CST	14793
HRP-conjugated secondary antibodies				
Antigen	Host species	Dilution	Source	Catolog number
Mouse IgG	Goat	1:20000	OriGene	ZB-2305
Rabbit IgG	Goat	1:20000	OriGene	ZB-2301
Rat IgG	Goat	1:20000	OriGene	ZB-2307
