# Supplementary material for: Both direct and indirect suppression of MCL1 synergizes with BCLXL inhibition in preclinical models of gastric cancer
Source: Cell Death Dis. 2025 Mar 12;16(1):170. doi: 10.1038/s41419-025-07481-8 (PMC11904182; doi:10.1038/s41419-025-07481-8)

Original Western Blot Data

Figure 1I

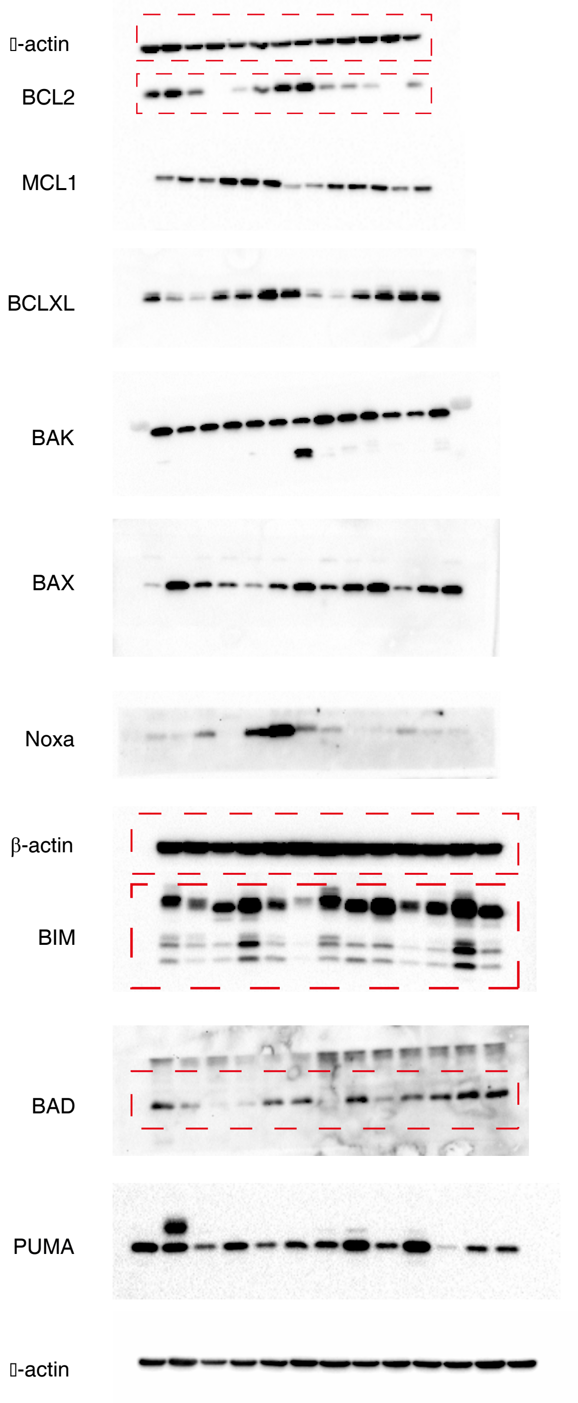

Original Western Blot Data

Figure S1A

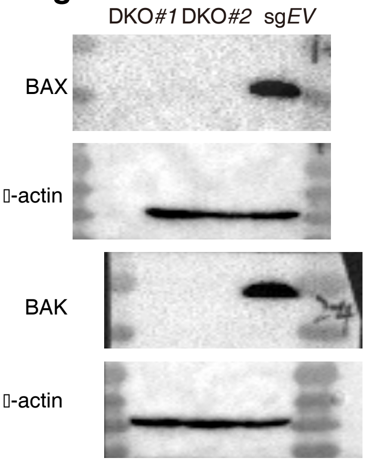

Figure S1B

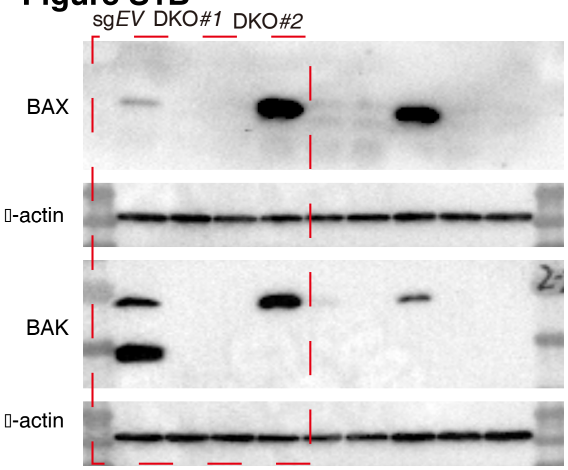

Figure S1H

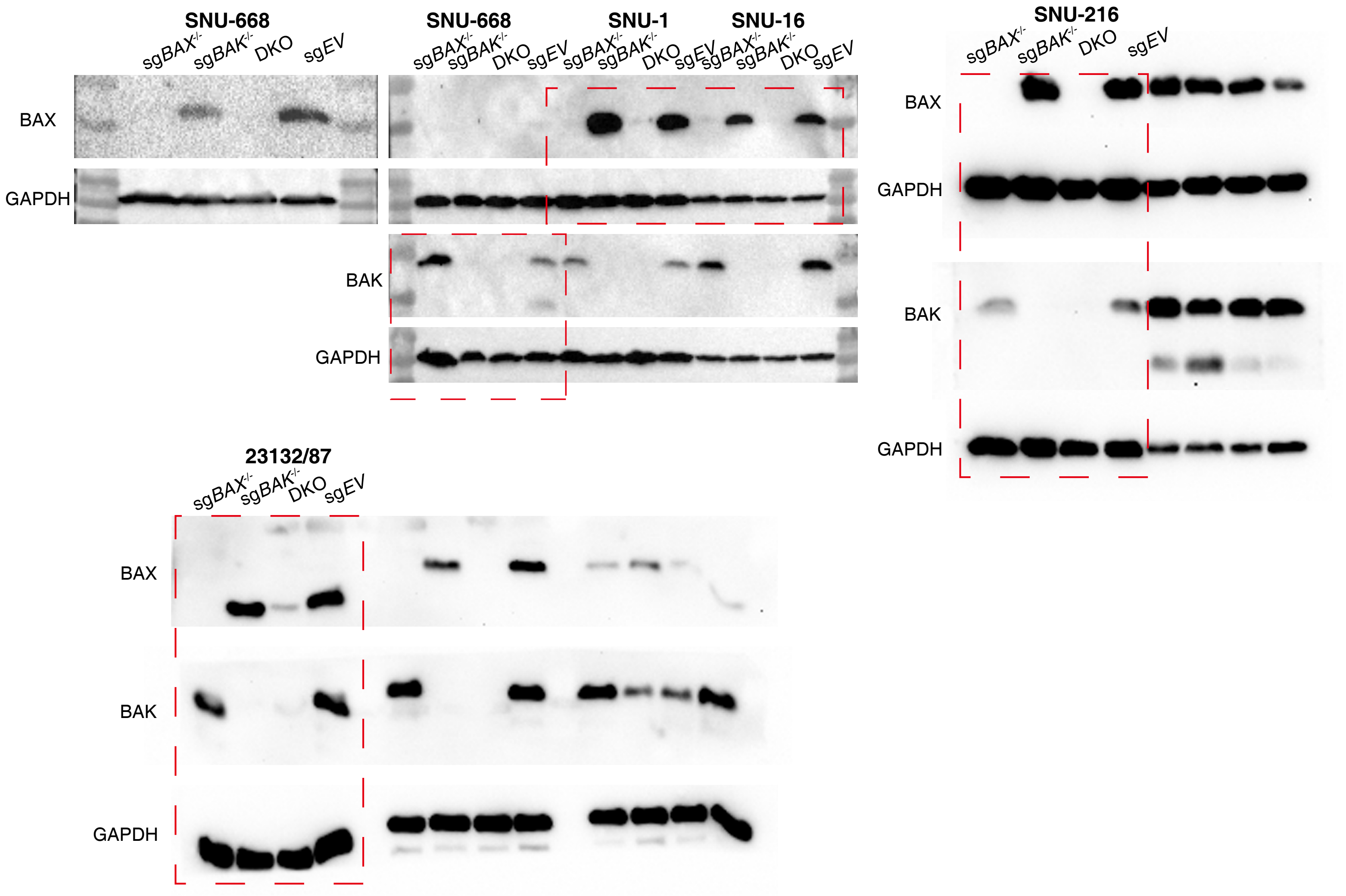

Original Western Blot Data
Figure S2C

23132/87

MCL1i
0 2 4 6 8

Caspase 3
Cleaved Caspase 3

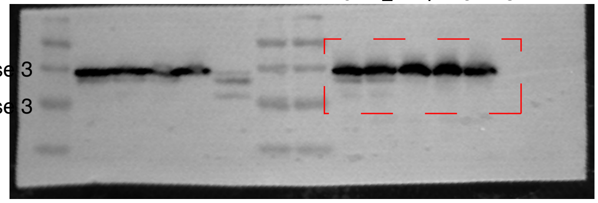

BCLXLi
0 2 4 6 8

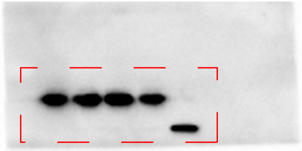

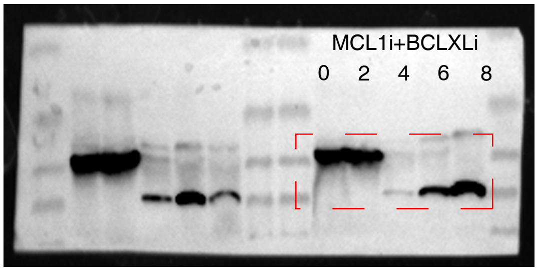

MCL1i
0 2 4 6 8

PARP
cleaved PARP

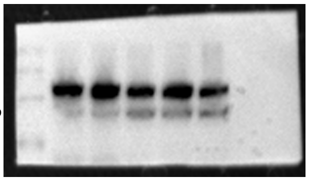

BCLXLi
0 2 4 6 8

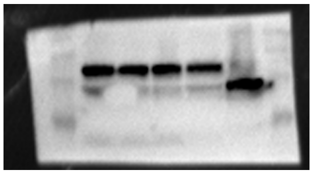

MCL1i+BCLXLi
0 2 4 6 8

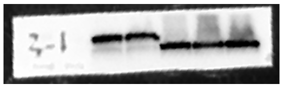

MCL1i

GAPDH

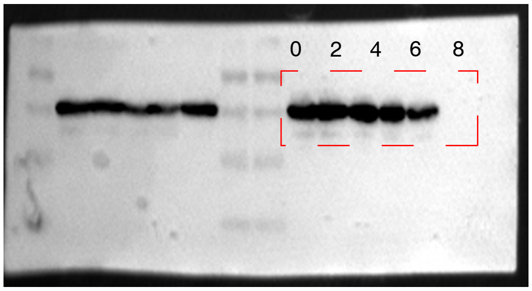

BCLXLi
0 2 4 6 8

GAPDH

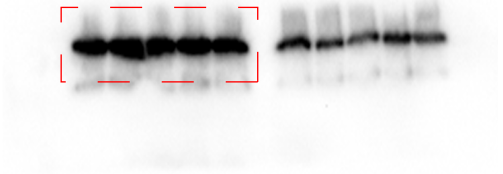

GAPDH

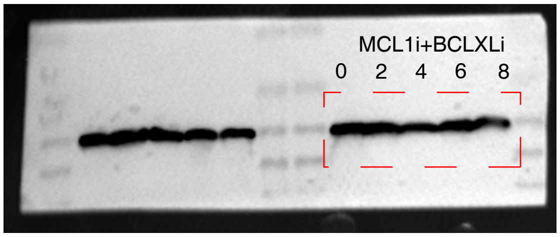

Original Western Blot Data
Figure S2C

SNU-668

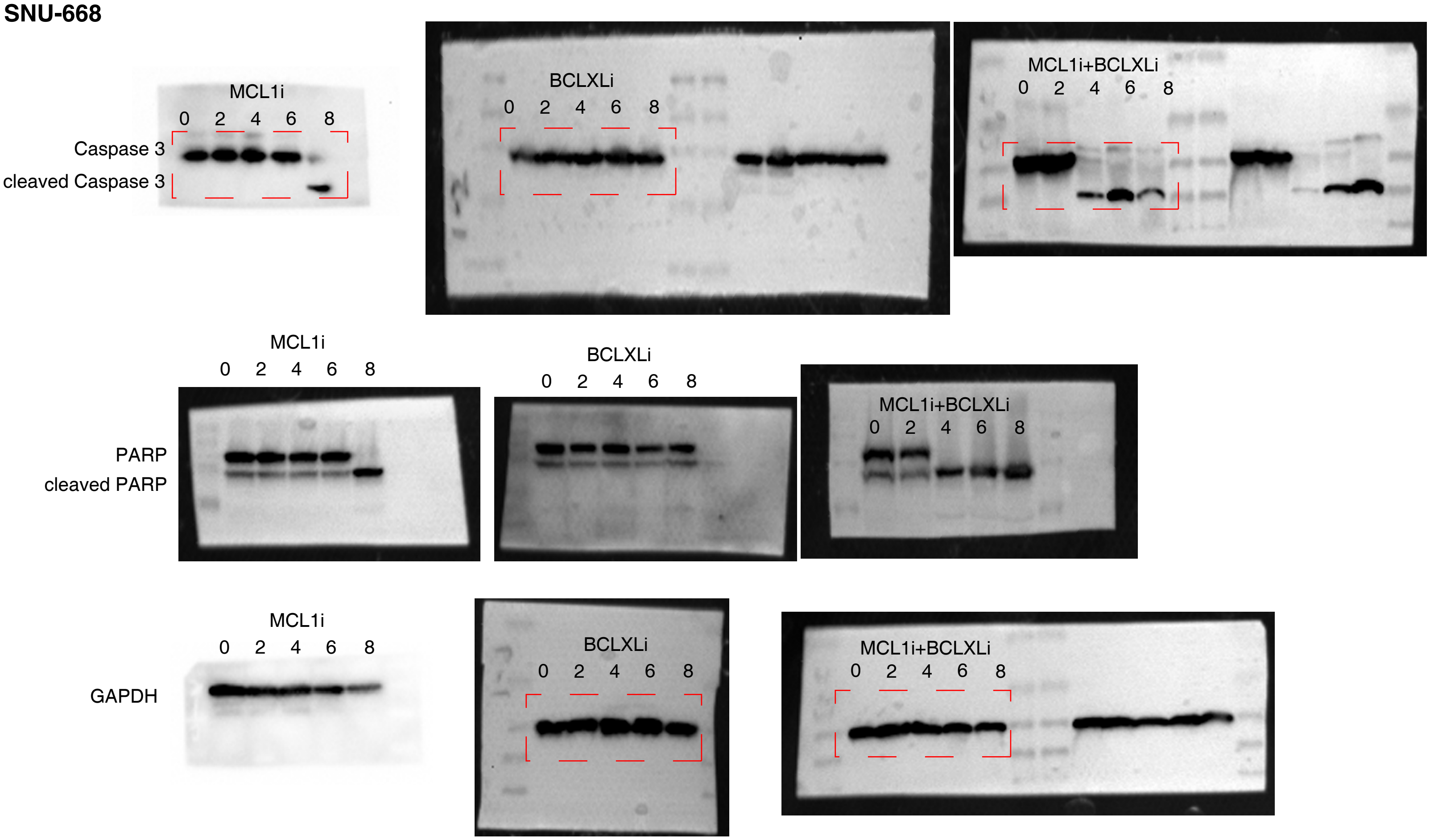

Original Western Blot Data

Figure 3A

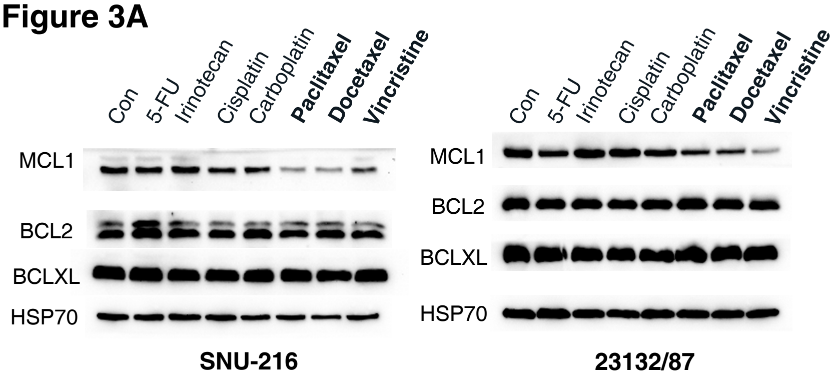

Figure 3B

SNU-216

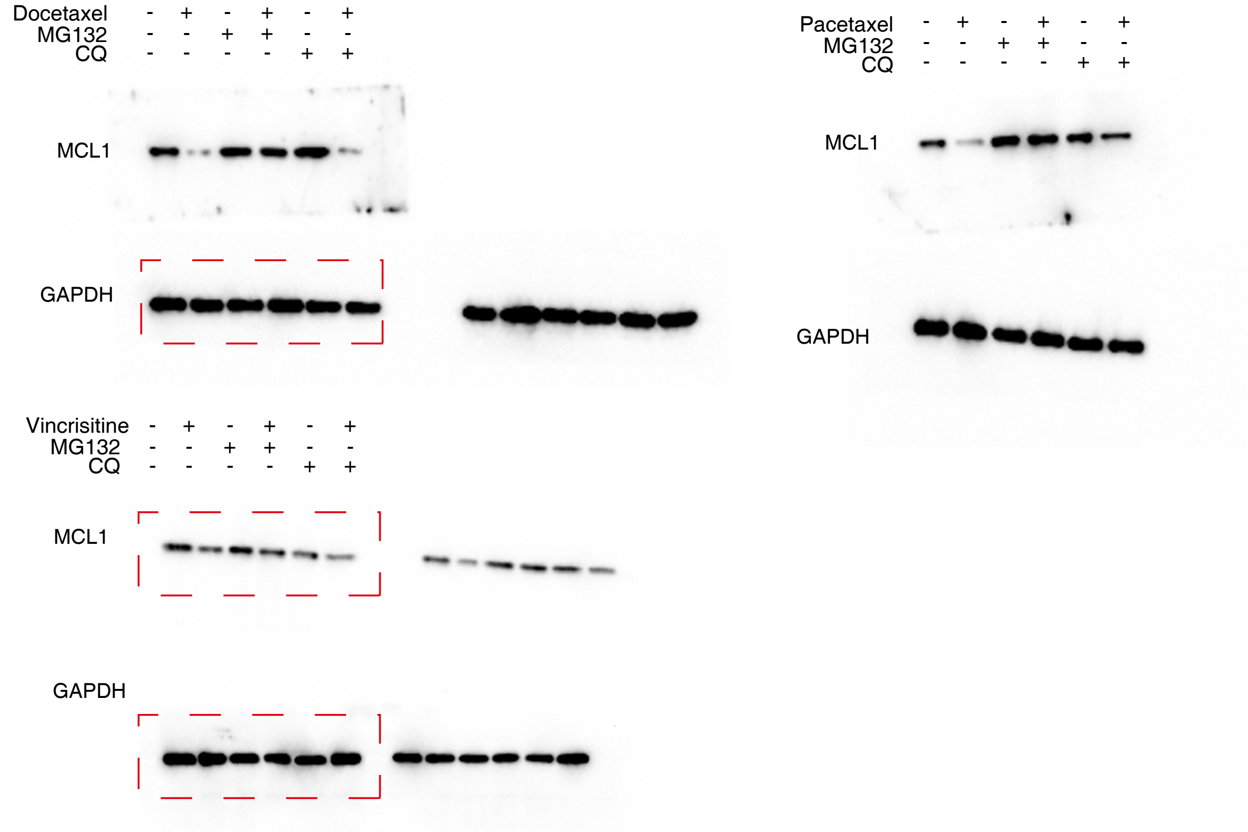

Original Western Blot Data

Figure 3C

23132/87

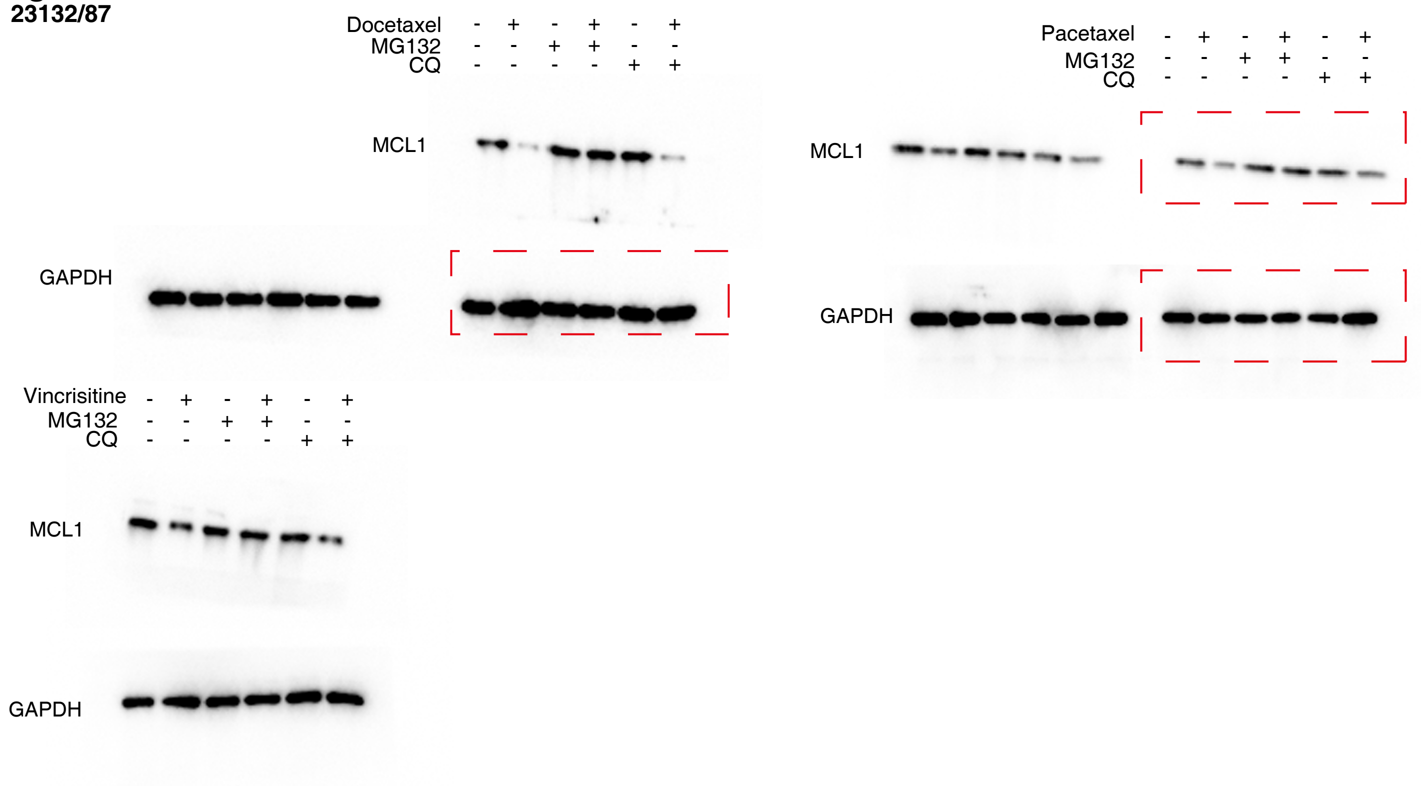

Figure 3E

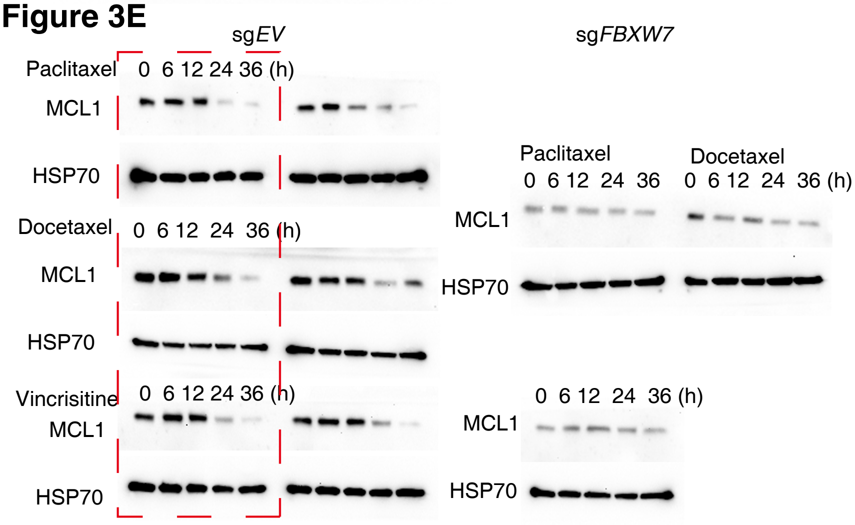

Figure 3F

MKN-1 (FBXW7 R465C)

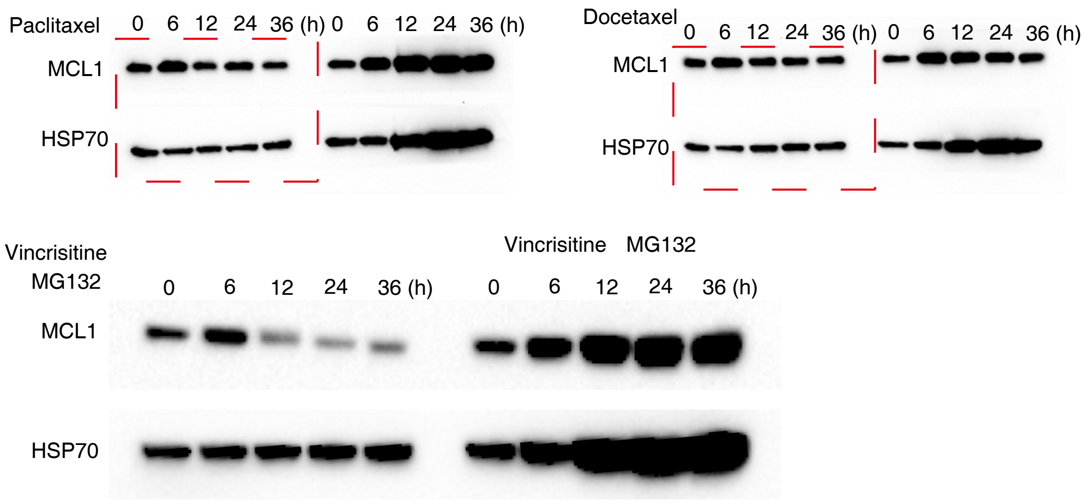

Original Western Blot Data

Figure S3A

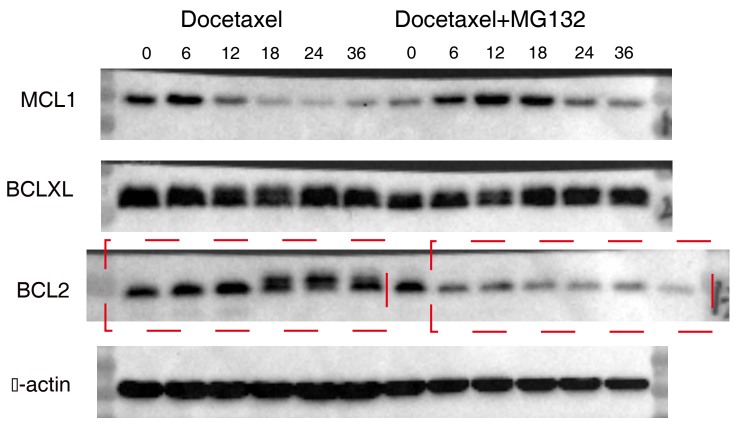

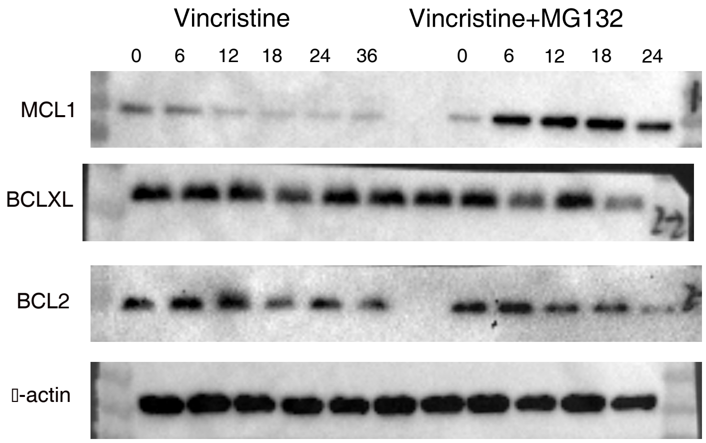

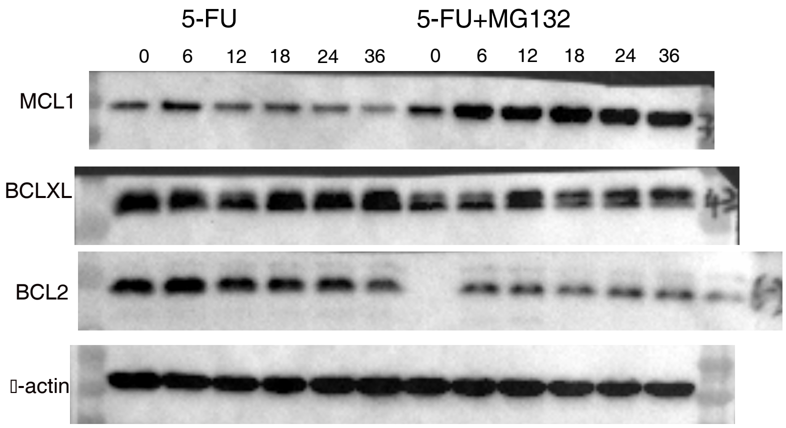

Original Western Blot Data

Figure 5A NCI-N87
(HER2-amplified)

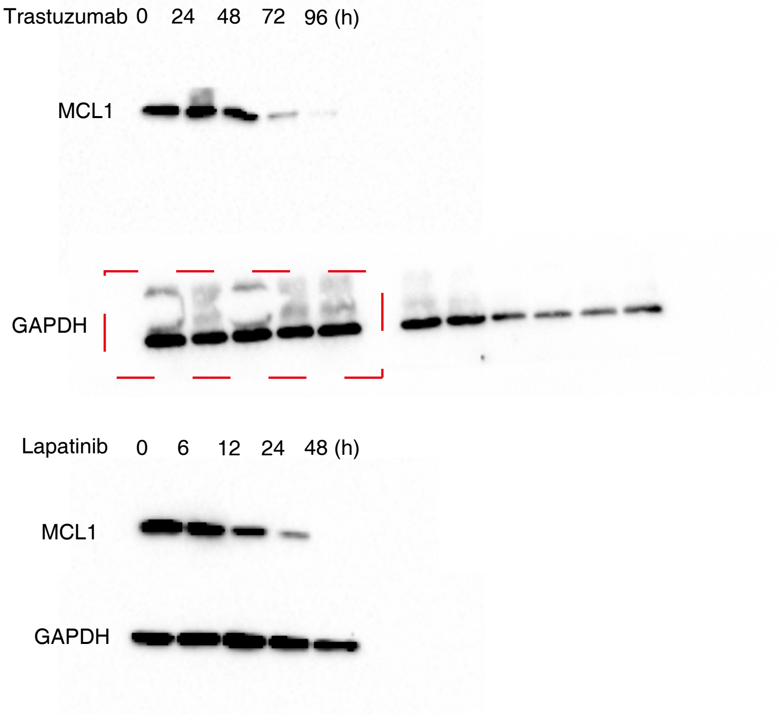

Figure 5B SNU-216
(HER2-amplified)

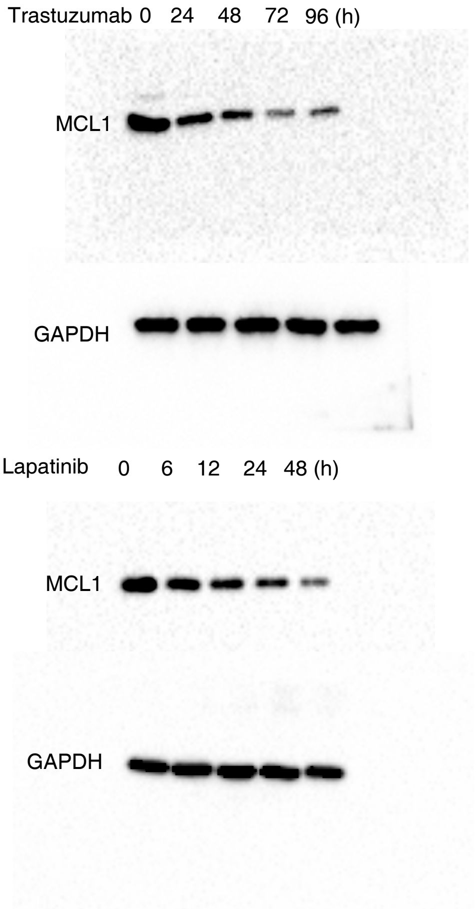

Figure 5C GCIY
(HER2-nonamplified)

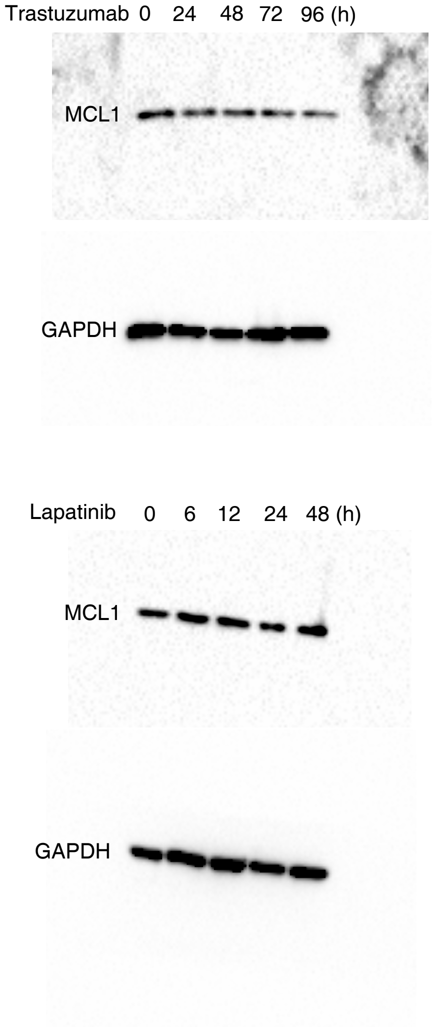

Figure 5D NCI-N87

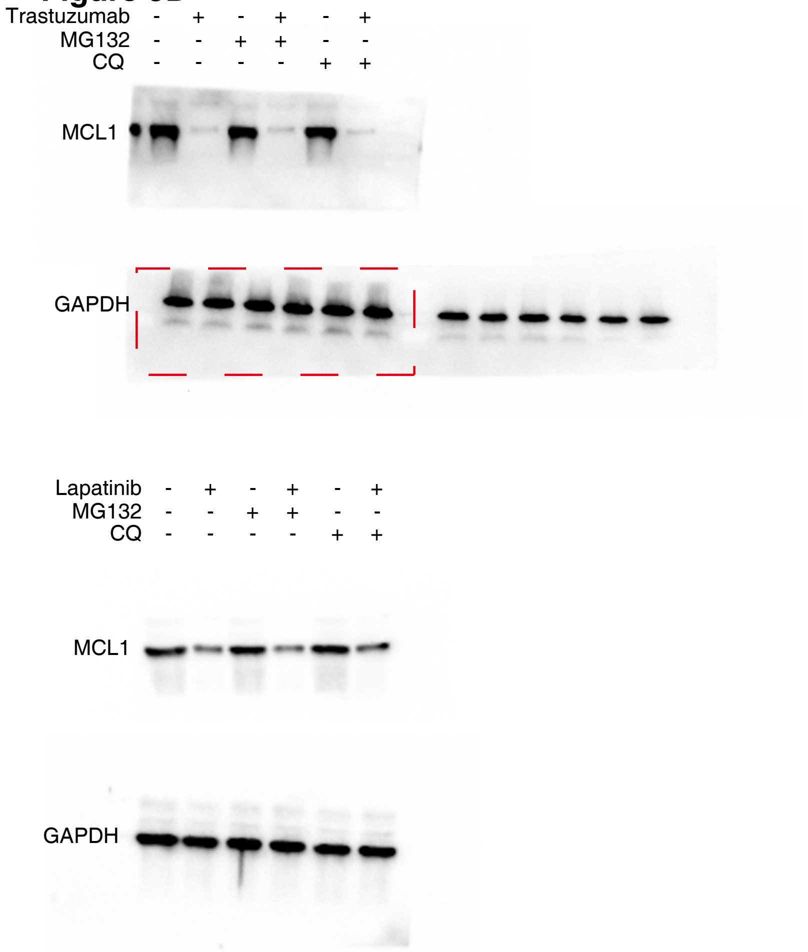

Original Western Blot Data

Figure S5A SNU-216

Trastuzumab	-	+	-	+	-	+
MG132	-	-	+	+	-	-
CQ	-	-	-	-	+	+

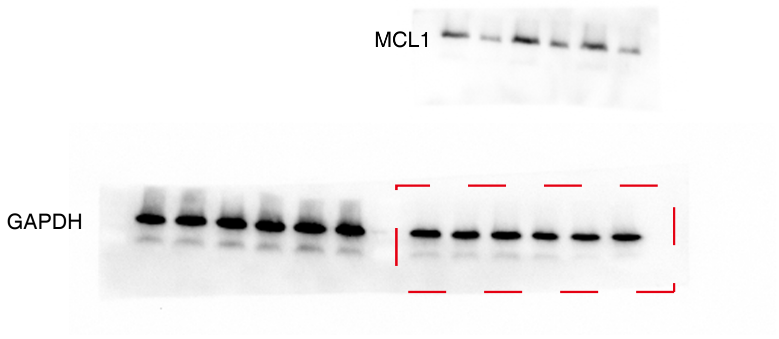

Lapatinib	-	+	-	+	-	+
MG132	-	-	+	+	-	-
CQ	-	-	-	-	+	+

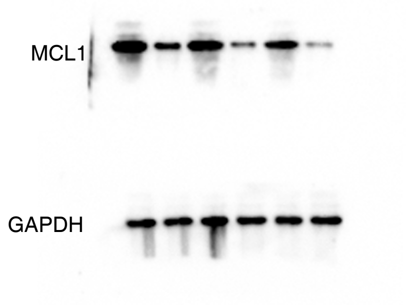

Original Western Blot Data

Figure 6C

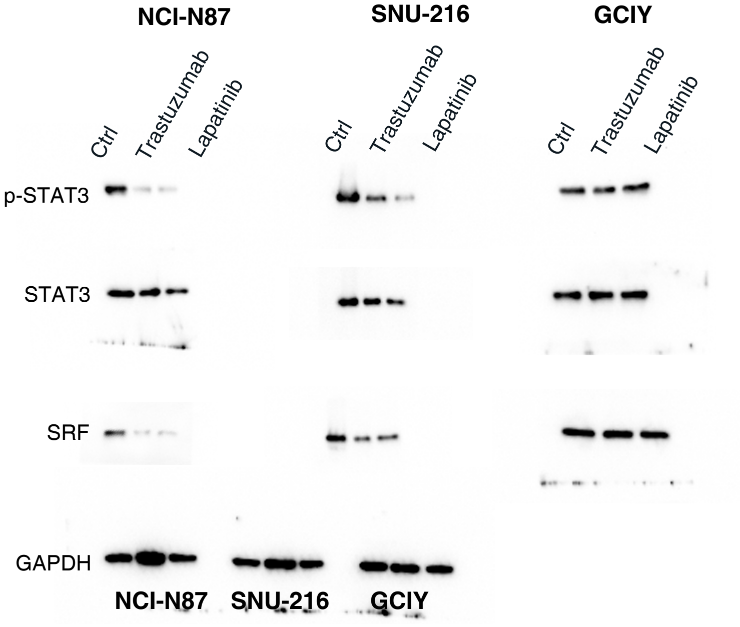

Figure 6G

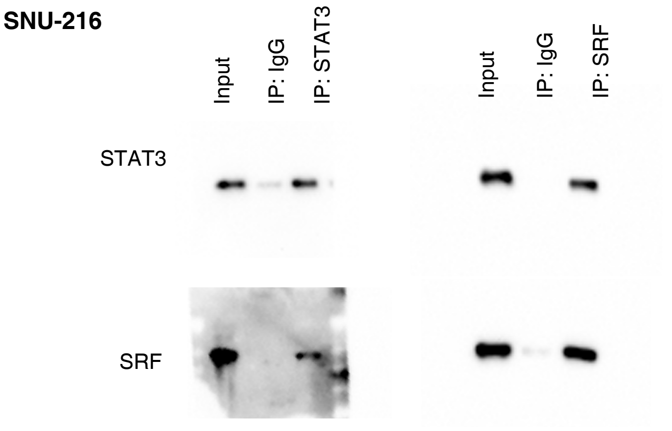

Figure 6H

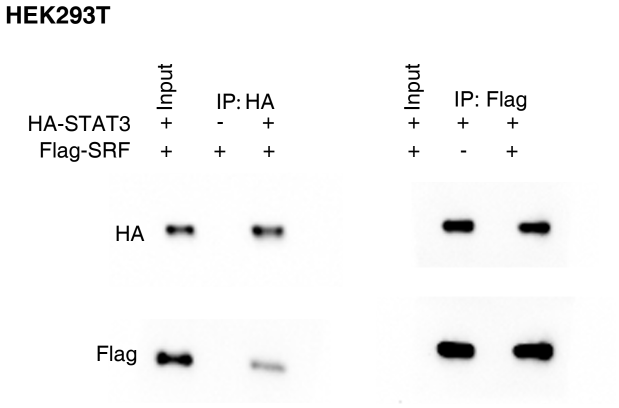

Figure 6I

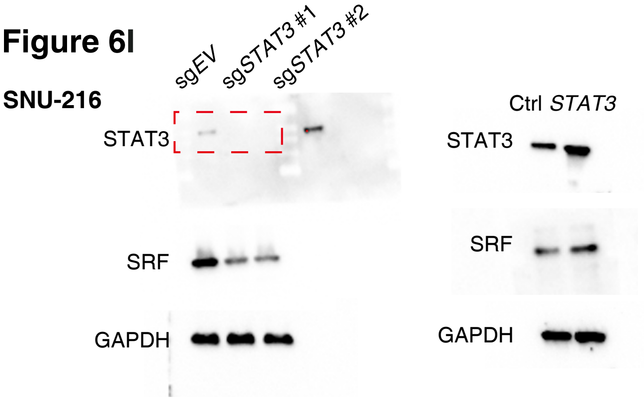

Figure 6J

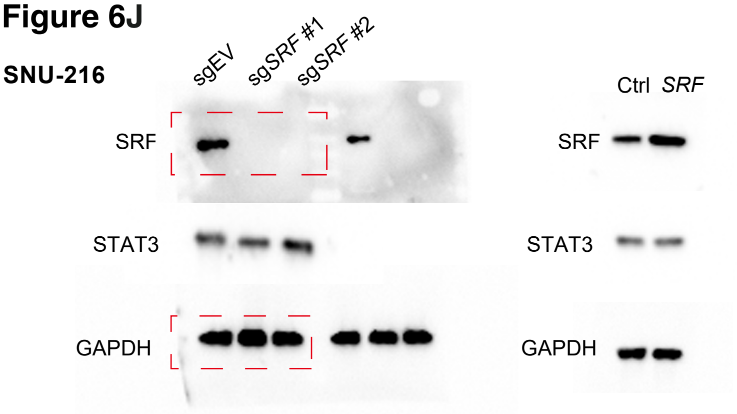

Original Western Blot Data

Figure S6A

SNU-216

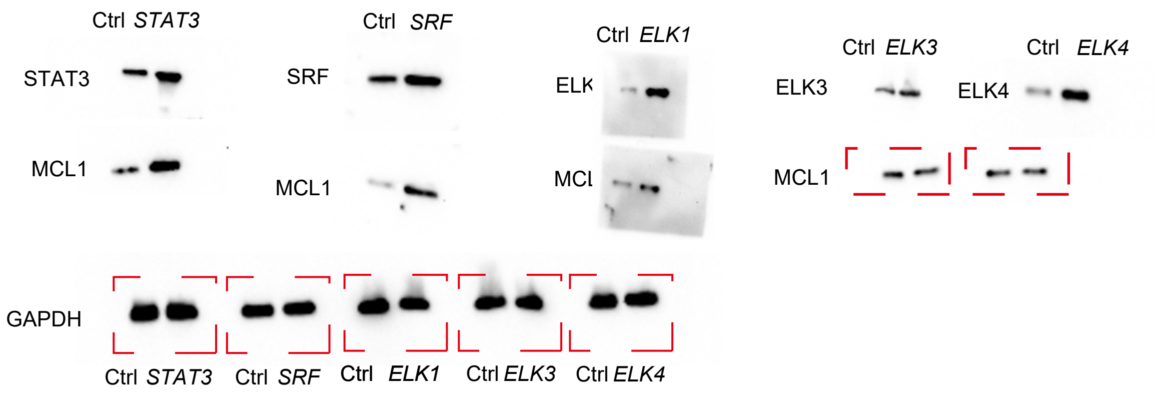

Figure S6D

SNU-216

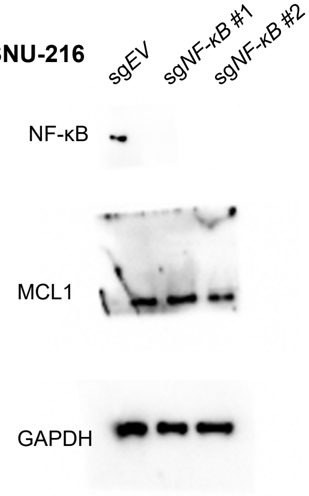

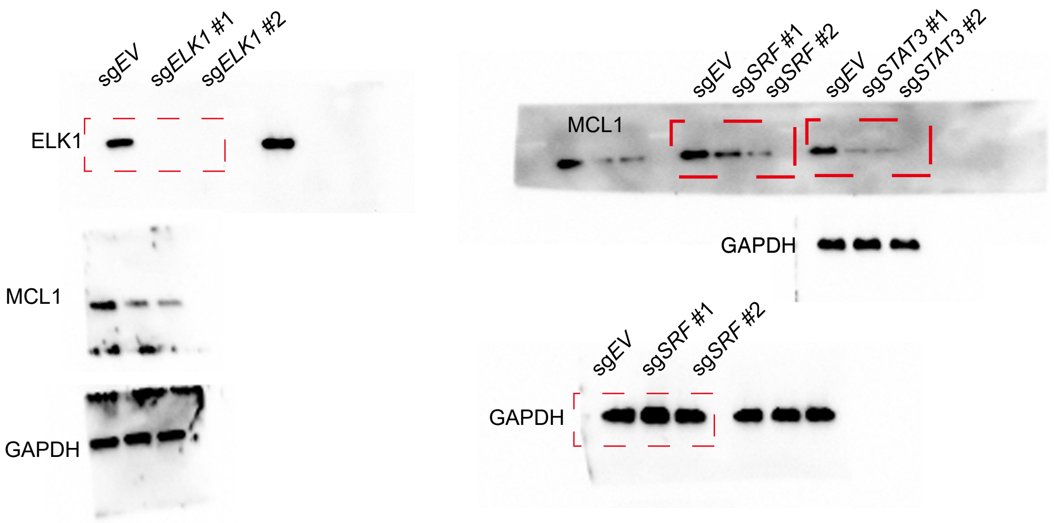

Figure S6H

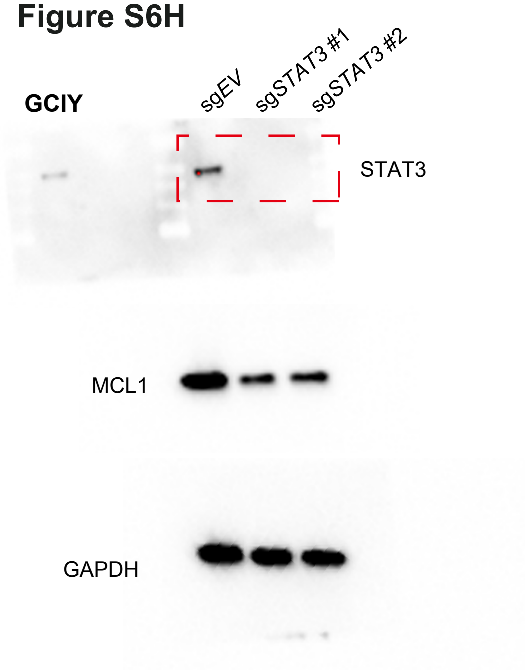

Original Western Blot Data

Figure S6I

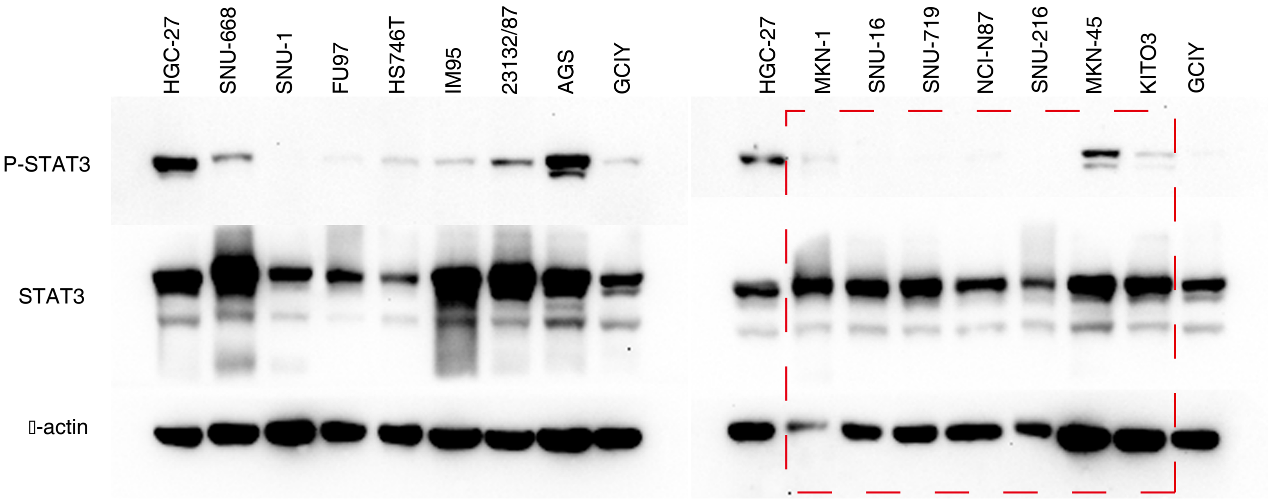

Original CHIP Data

Figure 6H

SNU-216

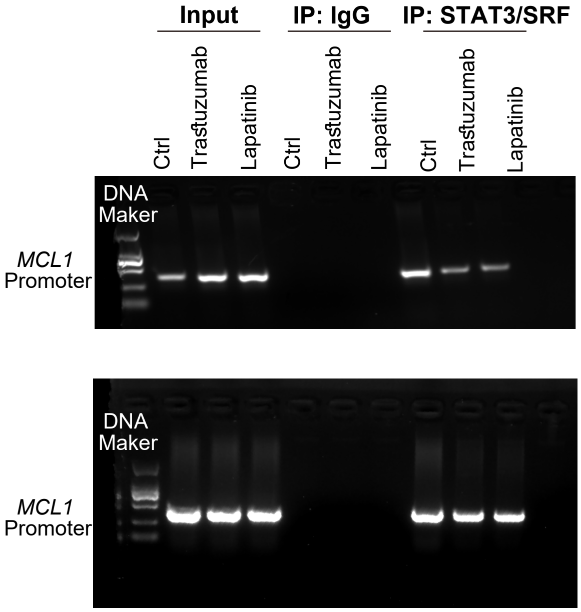

Figure 6K

SNU-216

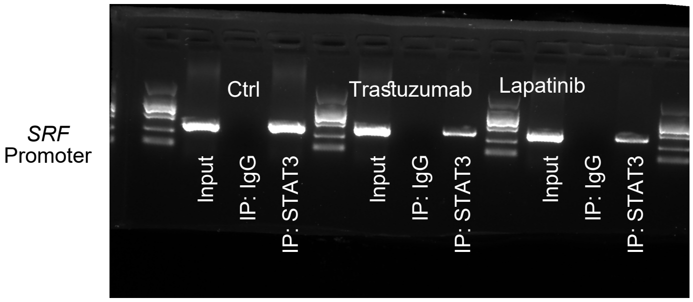

Figure 6L

SNU-216

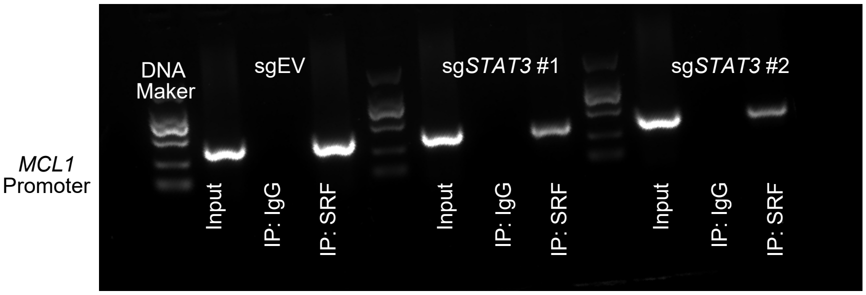

Supplement: Supplementary file 5 — Original Western Blot and ChIP PCR data [file 41419_2025_7481_MOESM5_ESM.pdf]
